# *BLADE‐ON‐PETIOLE* Genes Enable Genetic Bottleneck Mitigation Through Cross‐Species Repurposing of Floral Persistence Traits

**DOI:** 10.1002/advs.202517112

**Published:** 2026-03-28

**Authors:** Nan Xiao, Qianwen Lyu, Tinghao Zhang, Yupan Zou, Yue Xie, Dandan Yang, Cao Xu, Xiaozhen Huang

**Affiliations:** ^1^ Key Laboratory of Seed Innovation Institute of Genetics and Developmental Biology Chinese Academy of Sciences The Innovative Academy of Seed Design Chinese Academy of Sciences Beijing China; ^2^ University of Chinese Academy of Sciences Beijing China; ^3^ CAS‐JIC Centre of Excellence for Plant and Microbial Science (CEPAMS) Institute of Genetics and Developmental Biology Chinese Academy of Sciences Beijing China

**Keywords:** BLADE‐ON‐PETIOLE cofactors, floral organ abscission and senescence, phase separation, tomato, horticultural breeding

## Abstract

Continuous floral persistence caused by impaired petal abscission and senescence is generally detrimental in crops but beneficial in ornamental species, highlighting the species‐specific nature of breeding objectives. Although reduced genetic diversity and underutilization of many identified loci have imposed a profound bottleneck on molecular design breeding, divergent breeding goals across species enable strategic repurposing of genes previously considered detrimental. Here, we demonstrate the cross‐species functional repurposing of *BLADE‐ON‐PETIOLE* (*BOP*) genes in floral organ abscission and senescence and reveal the underlying molecular mechanism. Knockout of *SlBOP* genes resulted in defective petal abscission and delayed senescence, thereby compromising fruit appearance quality in tomato. In contrast, knockout of *PhBOP* genes in petunia produced non‐abscission and delayed senescence of corolla, markedly prolonging floral longevity and enhancing ornamental value. Mechanistically, we show that SlBOP transcriptional cofactors undergo phase separation and interact with the transcription factor TMF FAMILY MEMBER 1 (TFAM1) to form heterotypic biomolecular condensates that control abscission zone (AZ) formation, thereby orchestrating programmed floral organ abscission and senescence. Our findings illustrate that cross‐species functional analysis and knowledge transfer can provide a promising strategy to mitigate genetic bottlenecks and expand the toolkit for molecular design breeding.

## Graphical abstract

This study shows that BLADE‐ON‐PETIOLE (BOP) proteins regulate floral abscission and senescence in tomato by interacting with TFAM1 to form transcriptional condensates controlling the ARABIDOPSIS THALIANA HOMEOBOX GENE 1 (ATH1)‐mediated abscission pathway. Cross‐species transfer of BOP function extends flower longevity and improves corolla morphology in petunia, enhancing ornamental value and demonstrating how a detrimental trait can be repurposed to mitigate genetic bottlenecks in plant breeding.

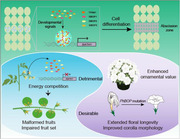

## 1 Introduction

Floral longevity and the timing of petal abscission are key determinants of ornamental value, directly influencing display duration and market attractiveness, particularly in cut‐flower species. However, the limited development of genetic and genomic research systems in ornamental species exacerbates the difficulty of gene identification and functional characterization. Given the pronounced divergence in breeding objectives across plant species, genetic variants that are deleterious in crops may hold untapped potential in ornamentals. Mutations that negatively affect agronomic performance, such as those altering leaf or floral architecture, may be advantageous in certain ornamental or specialty vegetable plants. For example, mutations in floral development genes often cause floral sterility in food crops, whereas in ornamental species like chrysanthemum, they induce double‐flower formation, and enhance aesthetic appeal [1]. Leaf curling, typically detrimental to yield in crops due to reduced photosynthetic efficiency [2], is a valued trait in cabbage because it confers a commercially desirable morphology [3]. Similarly, while timely organ abscission is essential for reproductive efficiency in crops [4, 5], delayed petal abscission is beneficial in ornamental flowers, as it prolongs floral longevity. These examples illustrate that redefining trait value according to specific breeding goals is essential for customized breeding strategies.

Abscission is a tightly regulated physiological process by which plant organs, such as senescent leaves, post‐pollination floral organs, and mature fruits or seeds, detach from the main body of the plant in response to developmental and environmental signals. This separation occurs at specialized structures known as abscission zones (AZs), where coordinated cellular differentiation, signaling, and enzymatic activities facilitate organ detachment [6, 7, 8]. Classically, the abscission process is divided into four phases: AZ cell formation, AZ cell activation, cell separation via cell wall remodeling, and protective layer formation [5]. Each phase is governed by intricate molecular networks involving phytohormones, transcriptional regulators, and a suite of hydrolytic enzymes [9, 10]. In Arabidopsis, the IDA‐HAESA (HAE)/HAESA‐LIKE 2 (HSL2) signaling module, in concert with SOMATIC EMBRYOGENESIS RECEPTOR‐LIKE KINASE (SERK) co‐receptors, activates MITOGEN‐ACTIVATED PROTEIN KINASE (MAPK) cascades to induce transcription factors such as BREVIPEDICELLUS (BP), KNOTTED 2/6‐LIKE (KNAT2/6), and AGAMOUS‐LIKE 15 (AGL15), which in turn regulate the expression of cell wall‐modifying genes [5, 11, 12]. Reactive oxygen species homeostasis, regulated by a secretory manganese superoxide dismutase MSD2, acts upstream of the IDA pathway through abscisic acid (ABA), and nitric oxide signals [13]. DNA BINDING WITH ONE FINGER (DOF) transcription factors, DOF4.7 and CYCLING DOF FACTOR 4 (CDF4), and the interaction between DOF4.7 and ZEAXANTHIN EPOXIDASE 2 (AtZFP2), **a transcription factor that suppresses floral organ abscission,** provide further layers of regulatory mechanism [14, 15, 16]. BLADE‐ON‐PETIOLE (BOP) transcriptional co‐factors, members of the NONEXPRESSOR OF PATHOGENESIS‐RELATED GENES 1 (NPR1) family, are essential for initiating AZ formation. Loss of *BOP* gene function leads to defective floral organ abscission in *Arabidopsis*, tobacco, tomato, and *Torenia fournieri* [17, 18, 19, 20, 21]. In *Arabidopsis*, BOP1/2 interact with TGACG‐BINDING FACTOR 1/4 (TGA1/4) to activate the expression of ARABIDOPSIS THALIANA HOMEOBOX GENE1 (ATH1), a TALE homeobox gene that is essential for floral organ AZ formation [22, 23, 24]. The synergistic functions of BOP‐ALOG complex have been demonstrated in *Arabidopsis* [25], tomato [18], pea [26], and *Torenia fournieri* [21, 27]. TfBOP2 interacts with TfALOG3, a member of the *Arabidopsis* LSH1, and *Oryza* G1 (ALOG) family, to promote corolla abscission in *Torenia fournieri* [21, 27].

AZ formation arises from the specialized differentiation of cells in response to abscission signals elicited by pollination or environmental stimuli, which requires precise, and rapid adjustment in cellular components. Characterized by rapid signal responsiveness and reversible biophysical states, liquid‐liquid phase separation (LLPS) enables spatially specific assembly and disassembly of proteins, and nucleic acids within cells. A growing number of studies have demonstrated that LLPS plays a crucial role in multiple biological processes by enhancing the efficiency of biochemical reactions, particularly those involved in gene expression, signal transduction, and stress responses [28, 29, 30]. Recent studies have revealed that transcriptional condensates formed via LLPS play a critical role in determining stem cell fate [31, 32, 33]. In this study, we demonstrate that SlBOP proteins undergo LLPS and form heterotypic condensates with the transcription factor TFAM1 to synergistically control programmed petal abscission and senescence in tomato. Furthermore, we successfully enhanced the ornamental traits of petunia by leveraging SlBOP orthologues. The cross‐species transfer of knowledge regarding *BOP* gene function exemplifies how mutations traditionally considered detrimental can be strategically repurposed for breeding.

## 2 Results

### 2.1 *SlBOP* Genes Program Floral Organ Abscission and Senescence in Tomato

While *BLADE‐ON‐PETIOLE* (*BOP*) genes are established regulators of floral organ abscission, the molecular basis of their function remains unclear. Given the contrasting breeding priorities for abscission in crops vs. ornamental plants, we investigated BOP‐mediated floral organ abscission in a crop context. To this end, we first monitored the temporal dynamics of petal abscission and senescence in wild‐type (WT) tomato plants. We selected floral buds expected to open the next day and performed pollination using an electric vibrator on the day of flowering to ensure successful pollination, and designated the day of flowering as 0 day after pollination (DAP0) (Figure S1A). Partial detachment and wilting of petals were observed at DAP3, progressing to complete abscission by DAP4 (Figure S1A,B). To quantify the timeline, we designated the petal state at DAP3 as indicative of both petal abscission and senescence.

**FIGURE 1:**
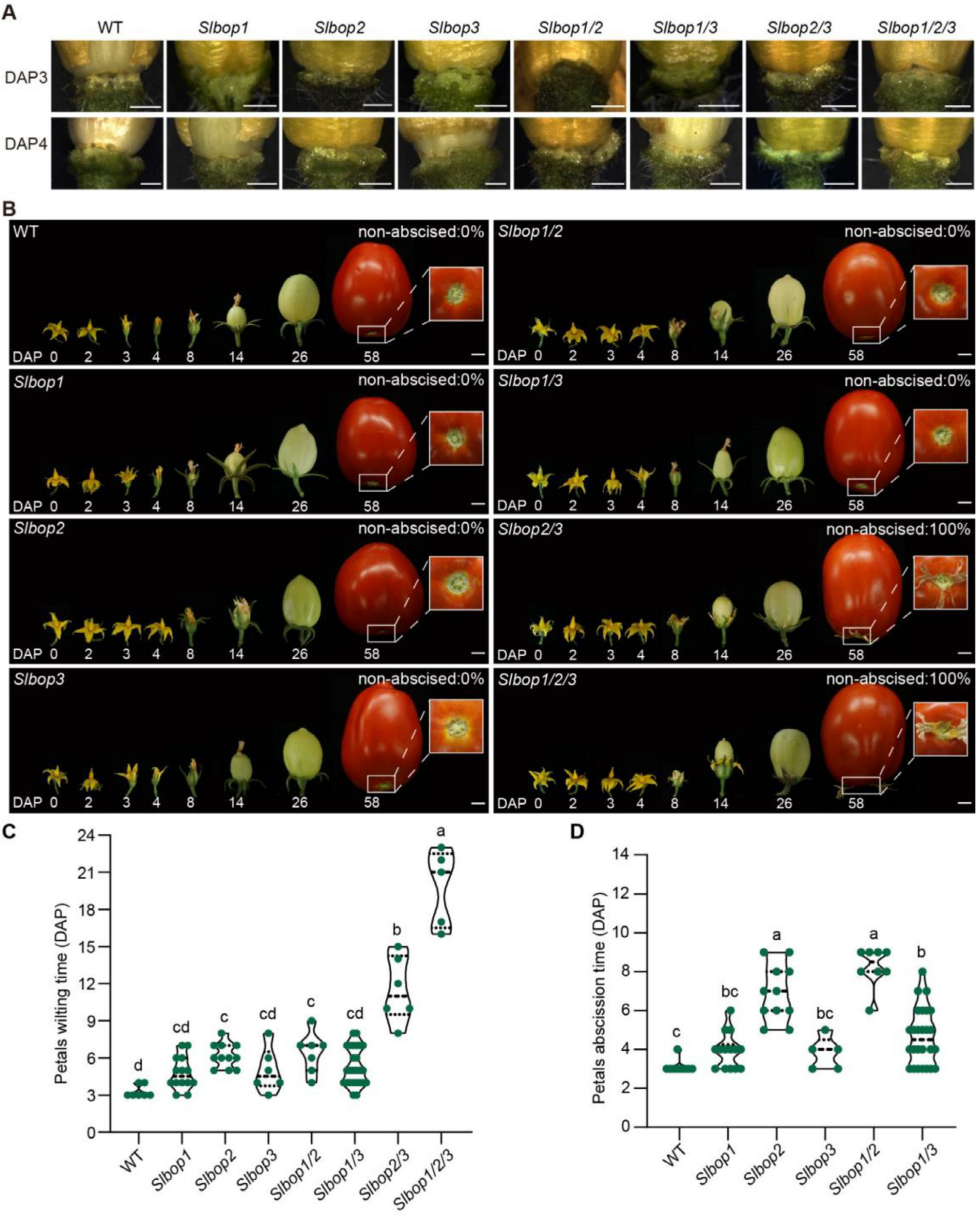
Programmed abscission and senescence of floral organs in *Slbop* mutants. (A) Stereomicroscope images of the boundary between the petal and the receptacle in flowers at 3 and 4 days after pollination (DAP3 and DAP4) from WT and *Slbop* mutants. Sepals of the flowers were forcibly removed. Scale bars, 1 mm. (B) Representative images showing the process of petals abscission and senescence in WT and *Slbop* mutants. Numbers indicate the days after pollination (DAP). Scale bars, 1 cm. (C,D) Quantification of days for the wilting (C) and abscission (D) of petals. DAP, day after pollination. Data are means ± SD (*n* = 7, 14, 11, 6, 7, 26, 6, and 5 for C; *n* = 9, 14, 11, 5, 8, and 26 for D), where n represents the number of biologically independent flowers. Statistical analysis was performed using one‐way ANOVA followed by Tukey's multiple comparisons test. Different letters indicate statistically significant differences among groups (*P* < 0.05).

We then conducted a systematic phenotypic analysis across *Slbop* single, double, and triple mutants. Specifically, *Slbop1* and *Slbop3* single mutants showed no significant differences in the timing of either petal abscission or senescence compared to the wild type (Figure 1A–D). The *Slbop1/3* double mutant exhibited a delay of approximately 2 days in petal abscission, while petal senescence occurred at a timing comparable to that of the wild type (Figure 1A–D). In contrast, both the *Slbop2* single mutant and the *Slbop1/2* double mutant exhibited a pronounced and significant delay in these processes, with both abscission and senescence postponed by approximately 4 and 3 days, respectively (Figure 1A–D). The *Slbop2/3* double mutant displayed a complete absence of petal abscission, coupled with a delay in petal senescence of approximately 8 days (Figure 1A–C). Notably, the *Slbop1/2/3* triple mutant also completely lacked petal abscission and showed an even more pronounced delay in senescence of approximately 16 days (Figure 1A–C). Collectively, these genetic analyses revealed a phenotypic redundancy among *SlBOP* genes, suggesting that petal abscission is predominantly regulated by SlBOP2 and SlBOP3, while all three members contribute to the regulation of petal senescence.

### 2.2 *SlBOP* Genes Promote AZ Formation by Regulating Cell Differentiation

**FIGURE 2:**
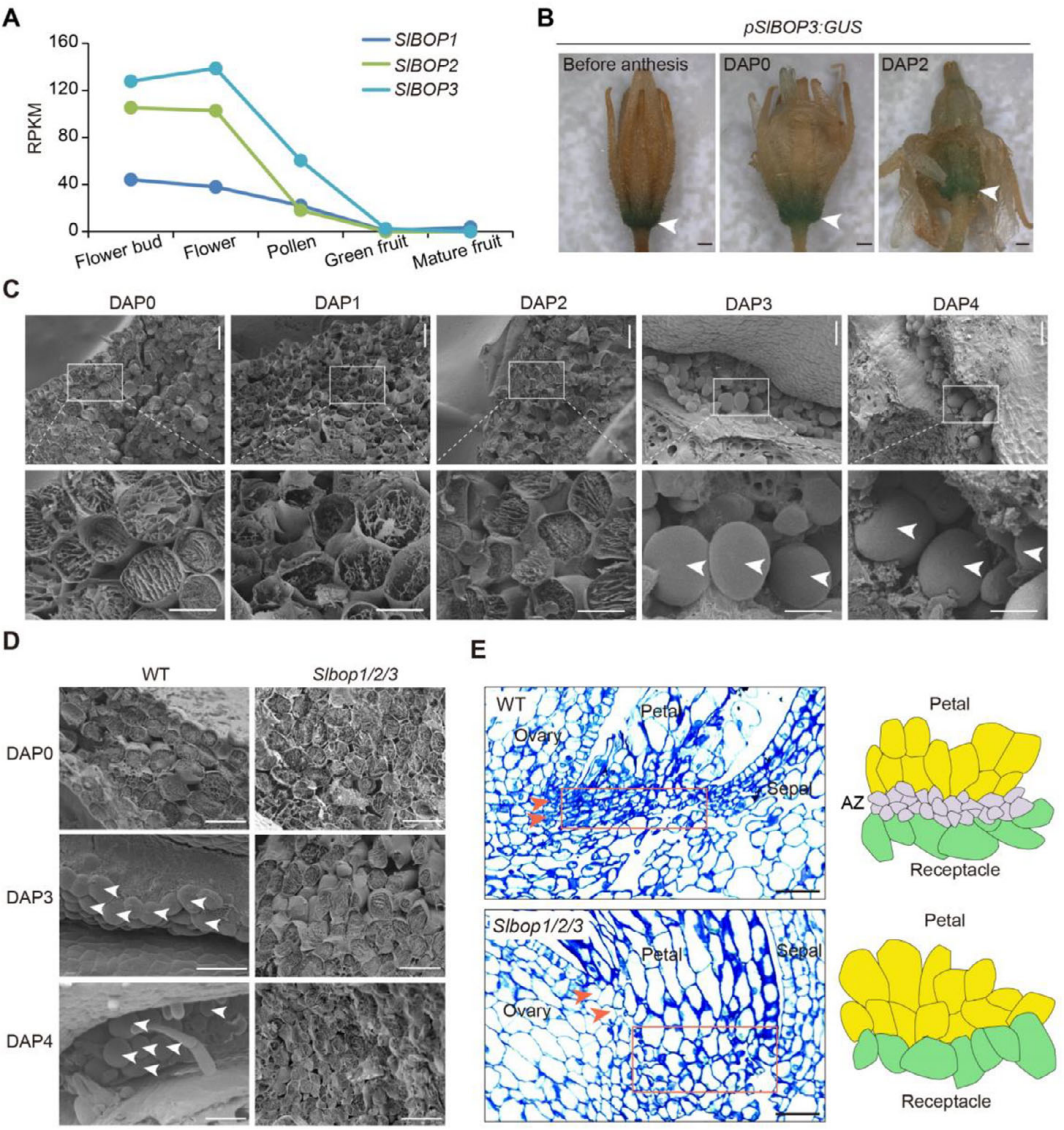
*SlBOP* genes are essential for the formation of petal AZ. (A) Normalized RNA sequencing read counts for *SlBOP1*, *SlBOP2*, and *SlBOP3* in flower bud, flower, pollen, green fruit, and mature fruit. (B) Histochemical GUS staining of flowers before anthesis, on the day of pollination (DAP0), and 2 days after pollination (DAP2) from *pSlBOP3:GUS* transgenic plants. Scale bars, 1 mm. White arrows indicate petal AZs. (C) Scanning electron micrographs of the fracture planes on the receptacle with petals removed in WT flowers at 0–4 days after pollination (DAP0‐4). The white arrows indicate the spherical elongated AZ cells. Scale bars, 50 µm. (D) Scanning electron micrographs of the fracture planes on the receptacle with petals removed in WT and *Slbop1/2/3* mutants at the DAP0, DAP3, and DAP4. The arrows indicate the spherical elongated AZ cells. DAP, days after pollination. Scale bars, 50 µm. (E) Longitudinal sections and schematics showing the boundaries of petal and other flower organs in WT and *Slbop1/2/3* mutants. Red arrows indicate cells adjacent to the ovary. Red boxes indicate AZ in WT and cells corresponding to WT AZ in *Slbop1/2/3* mutants. Scale bars, 50 µm.

AZ formation is a prerequisite for the programmed abscission of plant organs. In *Arabidopsis*, the AZ marker gene *At3g14380* shows no detectable expression in the *bop1/2* mutant, suggesting that BOP activity is required for AZ formation [34]. To test whether impaired petal abscission observed in *Slbop* mutants results from defects in AZ formation, we examined *SlBOP* gene expression patterns and performed cytological analyses in tomato. Transcriptomic data from the tomato expression atlas revealed that all three *SlBOP* genes exhibit relatively high expression levels in floral buds and flowers (Figure 2A). Semi‐quantitative RT‐PCR analysis confirmed the expression of *SlBOP* genes in AZ tissues collected from flowers at DAP 0 (Figure S2). To validate this, we generated *pSlBOP3:GUS* transgenic plants, in which the *SlBOP3* promoter drives *β*‐glucuronidase (GUS) expression. GUS staining showed strong activity at the junction between floral organs and the receptacle, a region anatomically coincident with the petal AZ (Figure 2B). These results support a regulatory role for *SlBOP* genes in AZ formation in tomato.

We next conducted cytological analysis of WT flowers using scanning electron microscopy (SEM) to investigate the cellular features associated with AZ formation. Specifically, we examined the split surface of the receptacle following either natural petal shedding or manual removal across developmental stages. From DAP0 to DAP2, ruptured cells were evident along the abscission plane, indicative of intercellular adhesion breakdown (Figure 2C,D). At DAP3, spherical and elongated cells began to emerge on the split plane, and by DAP4, these specialized cells densely populated the region where petals detach from the receptacle (Figure 2C,D). This morphological progression aligns precisely with the timeline of petal abscission phenotypes in WT plants (Figure S1B), suggesting that the presence of spherical elongated cells is a hallmark of abscission. In contrast, *Slbop1/2/3* triple mutant failed to exhibit these cellular features. SEM analysis revealed persistent ruptured cells on the split plane across all examined timepoints, without the emergence of spherical elongated cells (Figure 2D). Accordingly, the histological sectioning of the AZ region showed that, in WT flowers at DAP3, a distinct AZ structure was apparent, characterized by small, densely packed cells significantly shorter than adjacent cells on both sides (Figure 2E). Conversely, the putative AZ region in *Slbop1/2/3* mutant lacked these characteristic features; instead, the cells were elongated along the longitudinal axis, and morphologically indistinguishable from surrounding tissue, indicating a failure to specify and differentiate the AZ (Figure 2E). Together, these findings demonstrate that *SlBOP* genes regulate programmed floral organ abscission by promoting the differentiation of specialized cell layers that constitute the AZ.

### 2.3 SlBOP Proteins Undergo Phase Separation

**FIGURE 3:**
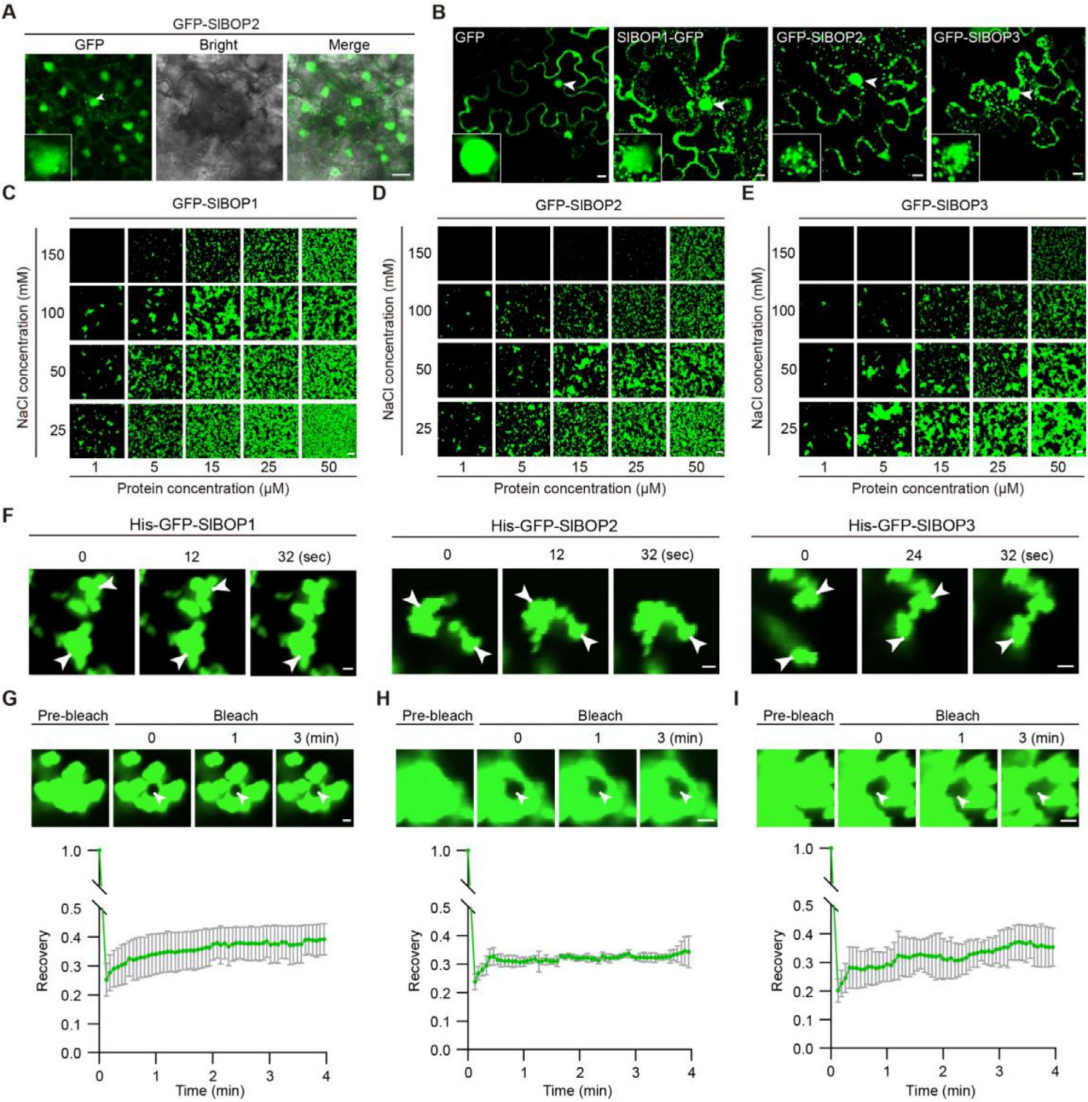
SlBOP proteins undergo phase separation. (A) Images showing the SlBOP2 condensates in the leaves of tomato transgenic plants expressing GFP‐SlBOP2. Scale bar, 10 µm. (B) Subcellular localization showing SlBOP condensates in the nucleus and cytoplasm of tobacco leaves. Scale bars, 10 µm. (C–E) Phase diagram of GFP‐SlBOP1 (C), GFP‐SlBOP2 (D), and GFP‐SlBOP3 (E) under different combinations of protein and NaCl concentrations. Scale bars, 20 µm. Three independent experiments with similar results were performed. (F) Representative images showing the fusion of GFP‐SlBOP1 (left), GFP‐ SlBOP2 (middle), and GFP‐SlBOP3 (right) droplets. Protein concentration, 15 µM. NaCl concentration, 50 mM. Scale bars, 1 µm. (G–I) Representative images and quantification data of FRAP analysis for GFP‐SlBOP1 (G), GFP‐SlBOP2 (H), and GFP‐SlBOP3 (I). Data are means of three independent FRAP events. Scale bars, 1 µm.

Although genetic analyses have established roles for *BOP* genes in floral organ abscission, the underlying functional properties of BOP proteins remain largely unexplored. To address this, we analyzed the protein sequences of SlBOPs and found that all three proteins harbor two typical intrinsically disordered regions (IDRs) (Figure S3A), a hallmark of proteins capable of phase separation [28]. One IDR is located within the DUF domain, while the other resides in the C‐terminal region (Figure S3A). Additionally, the BTB/POZ domain of SlBOP proteins harbors a putative redox‐sensitive region (Figure S3B). These features are reminiscent of NPR1, which undergoes salicylic acid‐induced condensation [35], implying that SlBOP proteins may form biomolecular condensates. To test this in planta, we generated tomato transgenic lines expressing GFP‐SlBOP2 proteins, which showed punctate distribution in both the nuclei and the cytoplasm of tomato cells (Figure 3A; Figure S4A). Consistently, transient expression of GFP‐tagged SlBOP1, SlBOP2, and SlBOP3 in tobacco leaves showed similar punctate patterns in both the nucleus and cytoplasm (Figure 3B). These results suggest that SlBOP proteins can form biomolecular condensates.

Given that the formation of biomolecular condensates is commonly driven by liquid‐liquid phase separation, we next examined the phase separation behavior of SlBOP proteins in vitro. To this end, GFP‐fused SlBOP proteins were recombinantly expressed and purified from *E. coli* (Figure S4B), followed by phase separation assays under varying protein and salt concentrations. All three SlBOP proteins exhibited phase separation behavior, forming irregular filamentous condensates that remained stable over time (Figure S4C). Phase diagrams revealed that both the size and density of condensates increased with protein concentration at a constant salt level, whereas increasing salt concentration reduced condensate formation at constant protein levels (Figure 3C–E), indicating that the phase separation behavior of SlBOP proteins is sensitive to both salt and protein concentrations. Notably, SlBOP1 formed visible condensates at as low as 5 µM protein concentration under near‐physiological salt conditions (150 mM NaCl), while SlBOP2 and SlBOP3 required higher thresholds (25 and 50 µM, respectively) (Figure 3C–E). Furthermore, under identical buffer conditions, SlBOP1 formed larger and denser condensates than SlBOP2 or SlBOP3 (Figure 3C–E), suggesting a stronger intrinsic propensity for phase separation and reduced salt sensitivity.

To evaluate the dynamic properties of phase‐separated SlBOP proteins, we performed time‐lapse imaging and fluorescence recovery after photobleaching (FRAP). Time‐lapse microscopy revealed rapid fusion events among droplets, indicative of liquid‐like behavior (Figure 3F). Consistently, FRAP analysis showed partial fluorescence recovery (13.39%–18.98%) within a few minutes after bleaching (Figure 3G–I), further supporting the dynamic and fluid nature of SlBOP droplets. Collectively, these results demonstrate that SlBOP1, SlBOP2, and SlBOP3 undergo liquid‐liquid phase separation, each exhibiting distinct biophysical properties, likely reflecting their functional divergence in planta.

### 2.4 SlBOP Proteins Interact with TFAM1 to Form Heterotypic Condensates

**FIGURE 4:**
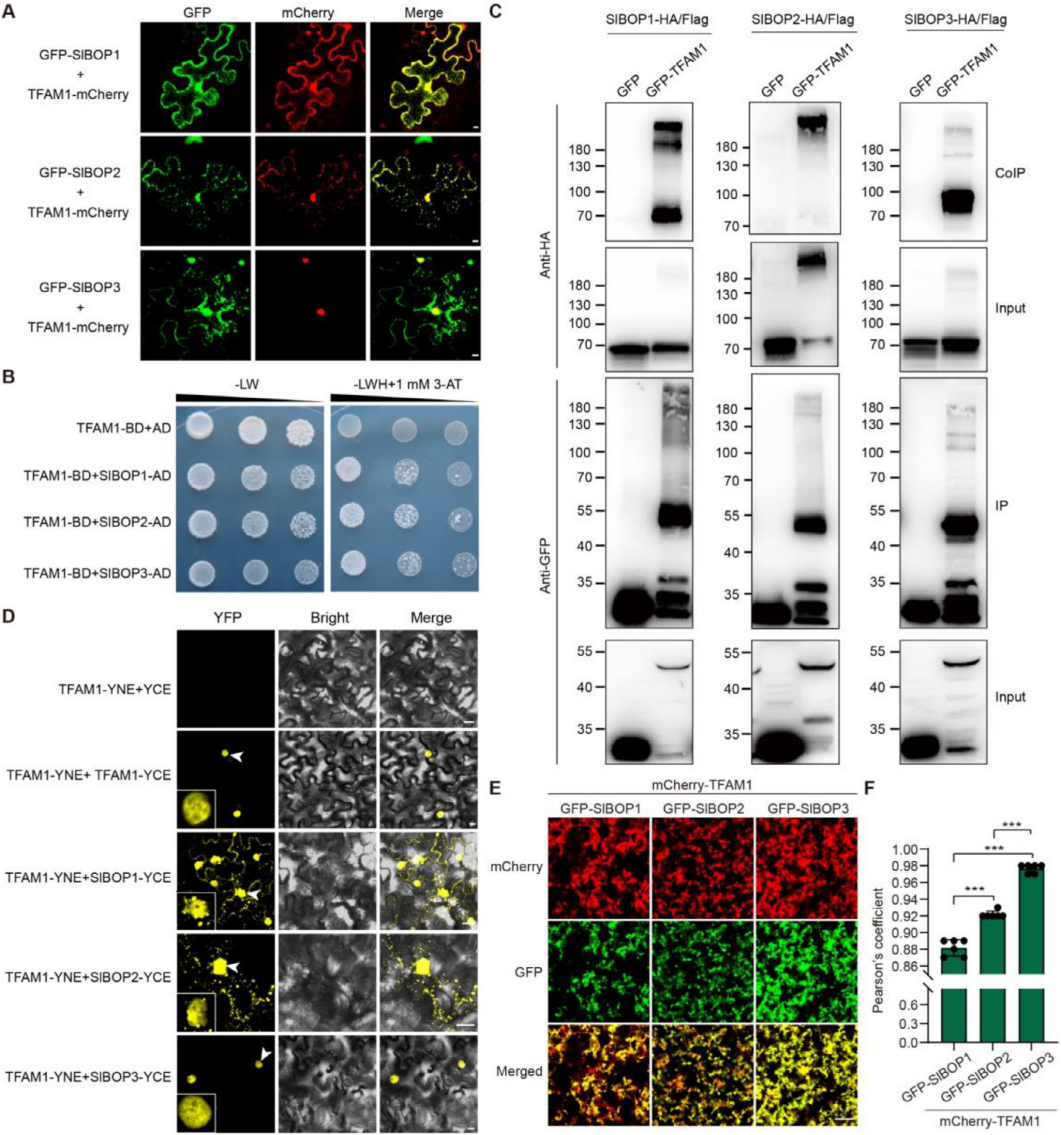
SlBOP proteins interact with TFAM1 to form heterotypic condensates. (A) Subcellular co‐localization analysis of TFAM1‐mCherry and GFP‐SlBOPs in tobacco leaves. Scale bars, 10 µm. (B) Yeast two‐hybrid assays showing that SlBOP1, SlBOP2, and SlBOP3 interact with TFAM1, respectively. Bait (BD) and prey (AD) constructs were co‐transformed into yeast cells as indicated. BD, DNA‐binding domain; AD, activation domain; −LW, nonselective medium minus Leu and Trp; −LWH + 3‐AT, selective medium minus Leu, Trp, and His supplemented with 1 mm 3‐amino‐1, 2, 4‐triazole (3‐AT). (C) Co‐immunoprecipitation (Co‐IP) assays showed that SlBOP1, SlBOP2, and SlBOP3 interact with TFAM1, respectively. GFP or GFP‐TFAM1 as a bait was immunoprecipitated with anti‐GFP beads, and SlBOP‐HA/Flag as prey was detected with anti‐HA. (D) BiFC assays in tobacco leaves show that SlBOP proteins interact with TFAM1, respectively. Scale bars, 10 µm. (E,F) Cross‐mixing phase separation reaction (E) and quantification of the intersection at 15 min (F) between recombinantly expressed mCherry‐TFAM1 and GFP‐SlBOP proteins. Protein concentration, 15 µM; NaCl concentration, 25 mM. Scale bar, 20 µm. Pearson's coefficient was calculated by ImageJ. Data are means ± SD (*n* = 6, 6, and 6, ^***^ *P* < 0.001, Student *t*‐test). Three independent experiments with similar results were carried out.

As transcriptional cofactors, BOP proteins often exert their functions through interactions with transcription factors [18, 21]. Consistent with this, subcellular localization analysis revealed that SlBOP proteins were co‐expressed with mCherry‐tagged TFAM1 (Figure 4A), an ALOG family transcription factor involved in abscission regulation [18]. Yeast two‐hybrid assays revealed that all three SlBOP proteins interact with TFAM1 (Figure 4B). To validate these interactions in vivo, we performed co‐immunoprecipitation (Co‐IP) assays by co‐expressing GFP‐tagged TFAM1 and HA/Flag‐tagged SlBOPs in tobacco leaves. The results demonstrated that three SlBOP proteins interact with TFAM1 in distinct forms. Specifically, SlBOP1 interacts as both oligomers and monomers, SlBOP2 primarily as oligomers, and SlBOP3 predominantly as monomers (Figure 4C). To visualize the interactions, we conducted bimolecular fluorescence complementation (BiFC) assays in tobacco leaves. All three SlBOP proteins form punctate homodimers both in the nucleus and the cytoplasm (Figure S5), while TFAM1 forms punctate homodimers specifically in the nucleus (Figure 4D). Notably, SlBOP1 and SlBOP2 interacted with TFAM1 to form condensates in both the nucleus and cytoplasm, whereas SlBOP3‐TFAM1 interaction resulted in nuclear condensate formation exclusively (Figure 4D). The localization of the interaction complex was consistent with their co‐localization (Figure 4A).

Given that TFAM1 can undergo phase separation to form heterotypic condensates with its homologs [32], we further investigated the biophysical properties of its interactions with SlBOP proteins in vitro. To exclude interference from endogenous plant proteins, we performed cross‐mixing phase separation assays using purified recombinant mCherry‐TFAM1 and GFP‐SlBOP proteins. After 15 min of incubation, incomplete but visible fusion events were observed between TFAM1 and SlBOP1, or SlBOP2, whereas SlBOP3 showed strong and homogeneous co‐condensation with TFAM1 in the same droplets (Figure 4E,F). Taken together, these findings demonstrate that SlBOP proteins interact with TFAM1 to form heterotypic biomolecular condensates via protein‐protein interactions and phase separation.

### 2.5 SlBOPs and TFAM1 Synergistically Control the Programmed Abscission and Senescence of Floral Organ

We next examined the role of ALOG proteins in AZ formation. To this end, we generated *pTFAM1:GUS* transgenic tomato plants expressing the *GUS* reporter gene under the control of the *TFAM1* promoter. GUS staining revealed that *TFAM1* is expressed at the junction between floral organs and the receptacle, which coincides with the AZ region (Figure S6A). Consistent with this, semi‐quantitative RT‐PCR analysis confirmed *TFAM1* expression in the AZ (Figure S6B). These results indicate that *TFAM1* exhibits an expression pattern similar to that of the *SlBOP* genes (Figures S2 and S6B). To further assess the subcellular localization of TFAM1, we developed transgenic tomato lines expressing GFP‐tagged TFAM1. GFP‐TFAM1 displayed punctate localization within the nuclei of AZ cells (Figure S6C), suggesting that TFAM1 may undergo phase separation to perform its function in the AZ. To investigate the functional role of TFAM1 in AZ formation, we examined cellular morphology at the petal–receptacle interface in *tfam1* mutant. Compared with wild type (WT), the appearance of spherical elongated cells on the split plane was delayed by approximately 1 day in *tfam1* mutant following petal removal (Figure S6D), indicating that TFAM1 is involved in promoting AZ formation.

**FIGURE 5:**
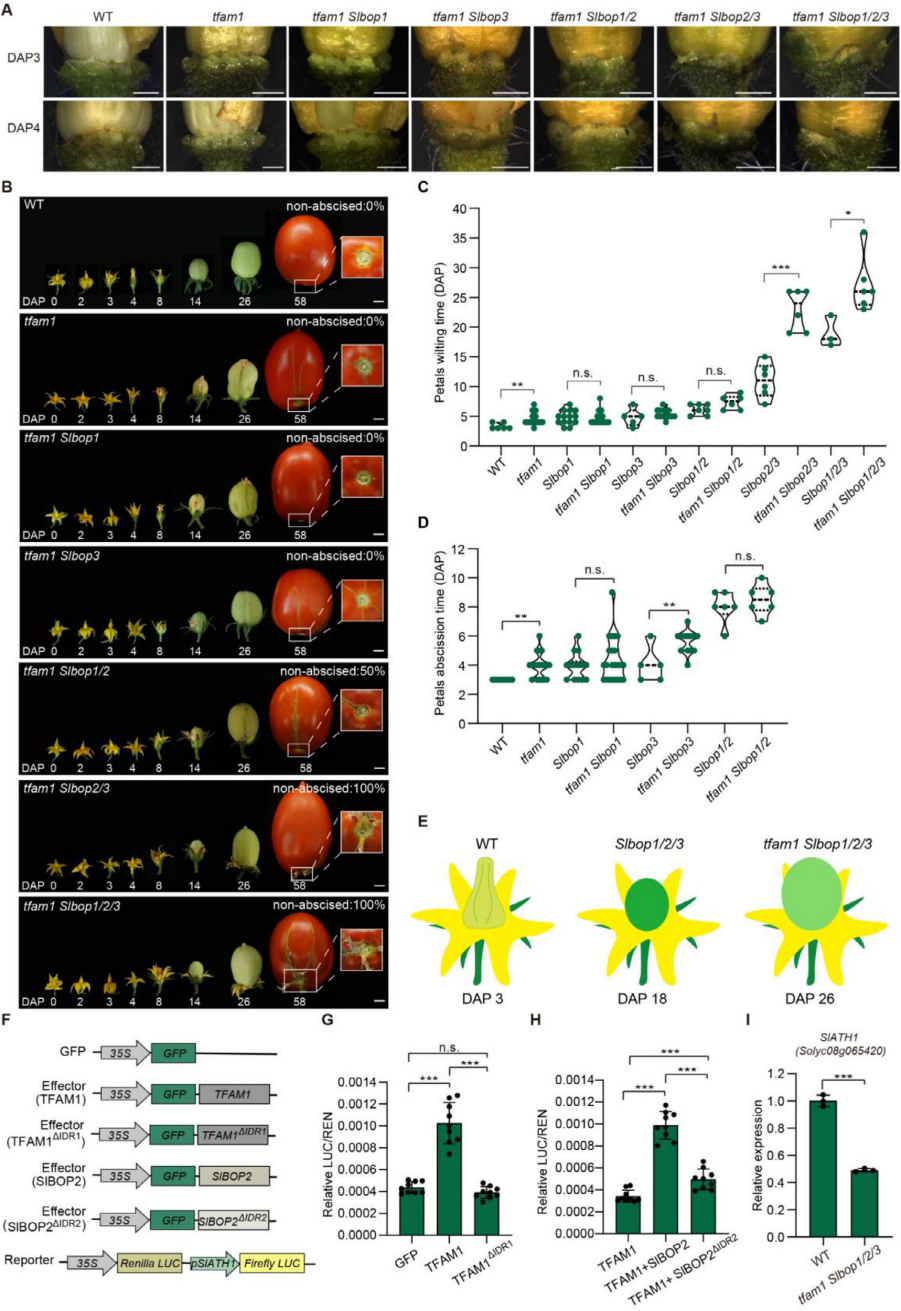
Programmed abscission and senescence of floral organs in *tfam1 Slbops* mutants. (A) Stereomicroscope images of the boundary between the petal and the receptacle in flowers at 3 and 4 days after pollination (DAP3 and DAP4) from WT and *tfam1 Slbop* mutants. Sepals of the flowers were forcibly removed. Scale bars, 1 mm. (B) Representative images showing the process of petals abscission and senescence in WT and *tfam1 Slbop* mutants. Numbers indicate the days after pollination (DAP). Scale bars, 1 cm. (C,D) Quantification of days for the wilting (D) and abscission (E) of petals. DAP, day after pollination. Data are means ± SD (*n* = 6, 19, 14, 23, 5, 13, 7, 6, 6, 6, 3, and 6 for C; *n* = 8, 19, 14, 23, 5, 13, 6, and 6 for D, ^*^ *P* < 0.05, ^**^ *P* < 0.01, ^***^ *P* < 0.001, Student *t*‐test), where n represents the number of biologically independent flowers. n.s., not significant. (E) Schematic illustrating the state of petals at the maximum duration of freshness retention for WT, *Slbop1/2/3*, and *tfam1 Slbop1/2/3* plants. (F–H) Schematic constructs (F) and transient dual‐luciferase reporter assays in tobacco (G,H). Relative luciferase activity indicates transcriptional activation of *SlATH1* by transcriptional condensates formed by TFAM1, SlBOP2, and the IDR‐deleted variants as indicated. Data are means ± SD (*n* = 9, ^***^P < 0.001, Student *t*‐test). n.s., not significant. Three independent experiments with similar results were carried out. (I) Relative expression of *SlATH1* in the abscission zone tissues of WT and *tfam1 Slbop1/2/3* mutant. *UBIQUITIN* served as an internal control. Data are means ± SD (*n* = 3, ^***^P < 0.001, Student *t*‐test). Three independent experiments with similar results were carried out.

To explore the genetic interactions between *BOP* and *ALOG* genes in regulating floral organ abscission and senescence, we conducted a systematic genetic analysis in tomato. Specifically, we generated a series of higher‐order mutants by crossing *tfam1* with various *Slbop* mutants. Phenotypic analysis revealed that compared to WT, *tfam1* single mutant exhibited an approximately 1‐day delay in petal abscission and senescence (Figure 5A–D), supporting its role in regulating programmed floral organ detachment. Given that *Slbop* mutants exhibit stronger phenotypes than the *tfam1* single mutant, we used *Slbop* mutants as reference backgrounds to assess the contribution of *TFAM1* in higher‐order combinations. The *tfam1 Slbop1* double mutant displayed similar abscission and senescence phenotypes to the *Slbop1* single mutant, while *tfam1 Slbop3* exhibited a 2‐day delay in petal abscission without significant changes in senescence compared to *Slbop3* (Figure 5A–D), suggesting a stronger genetic redundancy between TFAM1 and SlBOP3 than between TFAM1 and SlBOP1. Interestingly, while no significant difference in petal senescence was observed between *Slbop1/2* and *tfam1 Slbop1/2* mutants, approximately 50% of *tfam1 Slbop1/2* flowers showed persistent petals that failed to abscise until fruit ripening (Figure 5B,C). The remaining 50% resembled *Slbop1/2* in phenotype (Figure 5D), suggesting a partial genetic interaction. Furthermore, similar to the complete abscission defect observed in *Slbop2/3* and *Slbop1/2/3* mutants, the *tfam1 Slbop2/3*, and *tfam1 Slbop1/2/3* mutants also displayed a complete loss of petal abscission (Figure 5A,B). Notably, petal withering was further delayed by about 12 days in *tfam1 Slbop2/3* mutant compared to the *Slbop2/3* and about 8 days in *tfam1 Slbop1/2/3* mutant compared to the *Slbop1/2/3*, respectively (Figure 5A–C). Consequently, the petal freshness retention in *Slbop1/2/3* triple mutant is delayed by 16 days, and in *tfam1 Slbop1/2/3* quadruple mutant by 24 days, relative to the WT (Figure 5E). Together, these findings demonstrate that SlBOP1/2/3 and TFAM1 synergistically regulate both the abscission and senescence of tomato floral organs. The differential phenotypes of the higher‐order mutants reveal a hierarchical and partially redundant relationship among these genes. Specifically, TFAM1 exhibits the strongest functional association with SlBOP3, while SlBOP2 shows a closer relationship with SlBOP3 than with SlBOP1. These interactions suggest that SlBOP3 may serve as a molecular linker connecting the SlBOP cofactor complex with transcription factor TFAM1, enabling the formation of heterotypic condensates necessary for coordinated developmental regulation.

Previous studies have shown that TfALOG3 activates *TfATH1* expression to promote corolla abscission in *Torenia fournieri* [21]. Consistently, loss of ATH1 orthologues results in defective corolla abscission in *Torenia fournieri* [21] and *Arabidopsis* [24], indicating a conserved role of ATH1 in floral organ abscission. Based on these findings, we asked whether SlBOP‐TFAM1 condensates function through activation of a tomato *ATH1* orthologue. To test this, we first performed a BLAST search using AtATH1 and TfATH1 as queries to identify the tomato orthologue of ATH1. Phylogenetic analysis revealed that *Solyc08g065420* clusters with AtATH1 and TfATH1 (Figure S7), indicating it is the putative tomato ATH1 orthologue. Accordingly, *Solyc08g065420* was named as *SlATH1*. We next performed a series of transcriptional activity assays using a luciferase dual reporter system in tobacco leaves (Figure 5F). The results showed that TFAM1 activates *SlATH1* transcription, and that deletion of the IDR1 of TFAM1 abolished this effect (Figure 5G). Co‐expression of SlBOP2 further enhanced the transcriptional activation (Figure 5H); however, deletion of the IDR2 dramatically disrupted puncta formation of the SlBOP2 protein (Figure S8) and attenuated this enhancement (Figure 5H). These results suggest that IDR‐mediated phase separation is essential for the activity of SlBOP‐TFAM1 transcriptional condensates. Consistently, quantitative RT–PCR analysis revealed that *SlATH1* expression was significantly reduced in the *tfam1 Slbop1/2/3* quadruple mutant compared with the wild type (Figure 5I). Together, these results suggest that SlBOP‐TFAM1 transcriptional condensates regulate programmed floral organ abscission and senescence through the conserved ATH1‐mediated abscission pathway in tomato.

### 2.6 Cross‐Species Transfer of BOP Functions Enhances Ornamental Traits

**FIGURE 6:**
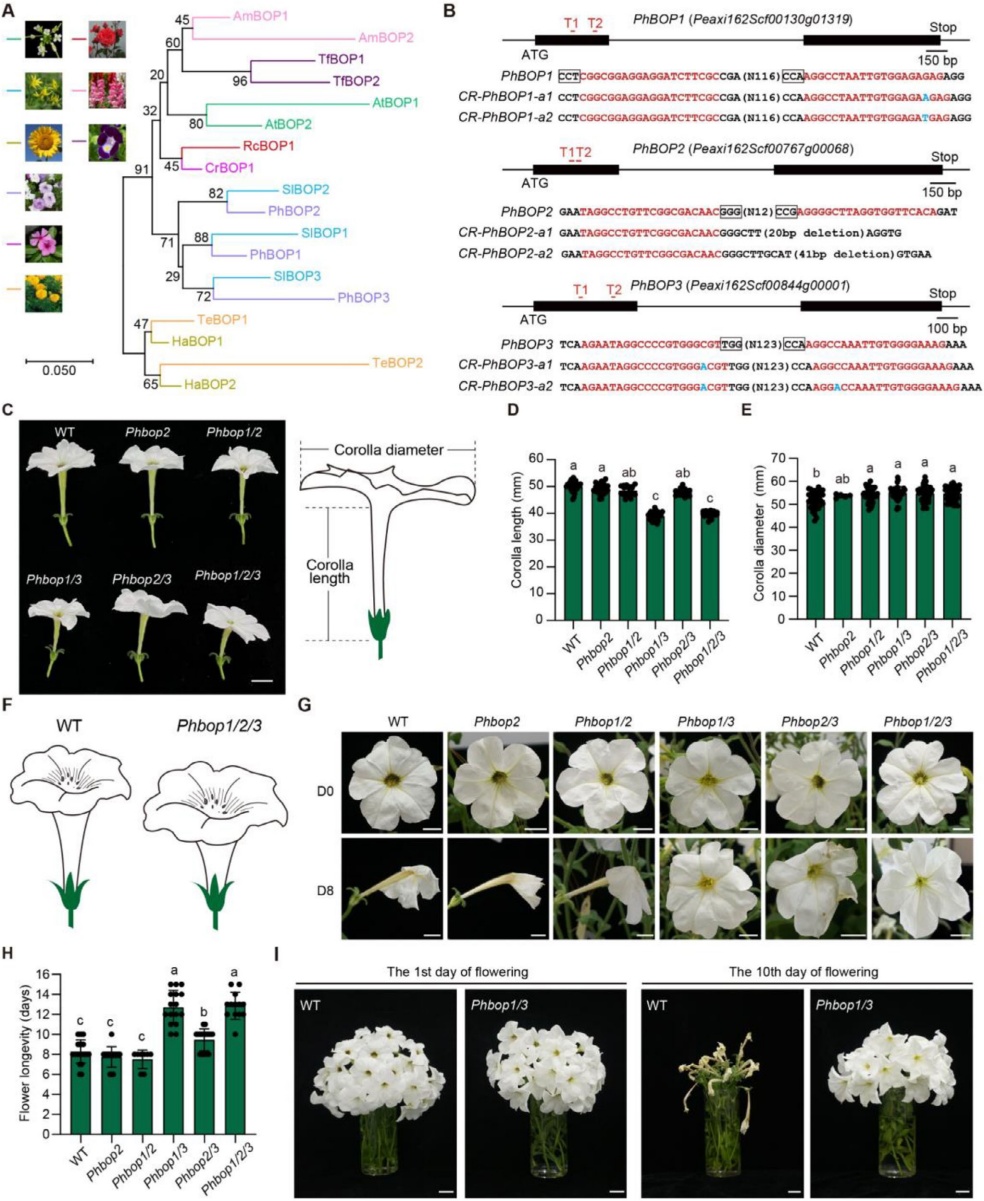
Mutations of *PhBOP* genes in petunia improve horticultural traits. (A) Maximum likelihood phylogenetic tree of BOP‐like proteins from *Arabidopsis thaliana* (*At*), *Solanum lycopersicum* (*Sl*), *Petunia hybrid* (*Ph*), *Catharanthus roseus* (*Cr*), *Tagetes erecta L*. (*Te*), *Rosa chinensis* (*Rc*), *Antirrhinum majus* (*Am*), *Helianthus annuus* (*Ha*), and *Torenia fournieri* (*Tf*). (B) Schematic (upper) indicating sgRNAs (red lines) and allelic information (bottom) for *CR‐PhBOP1*, *CR‐PhBOP2*, and *CR‐PhBOP3* mutants, respectively. The red font highlights sgRNA targets and black boxes indicate protospacer‐adjacent motif (PAM) sequences. (C–E) Schematic of representative images of corolla (C), quantification of corolla length (D) and diameter (E) for WT and *Phbop* mutants. Scale bars, 2 cm. Data are means ± SD (*n* = 31, 22, 19, 31, 18, and 23 for D, *n* = 33, 5, 46, 43, 36, and 41 for (F), where n represents the number of biologically independent flowers. Statistical analysis was performed using one‐way ANOVA followed by Tukey's multiple comparisons test. Different letters indicate statistically significant differences among groups (*P* < 0.05). F) Schematic diagrams showing the flowers of WT and *Phbop1/2/3* mutant. (G) Representative unpollinated flowers from WT and *Phbop* mutants at the day of flowering (D0) and 8 days after flowering (D8). Scale bars, 1 cm. (H) Quantification of the flower duration time of unpollinated flowers from WT and *Phbop* mutants. Data are means ± SD (*n* = 22, 15, 8, 16, 18, and 13), where n represents the number of biologically independent flowers. Statistical analysis was performed using one‐way ANOVA followed by Tukey's multiple comparisons test. Different letters indicate statistically significant differences among groups (*P* < 0.05). (I) Multiple detached flowers from WT, *Phbop1/3* mutants at the 1st day of flowering and the 10th day of flowering. Scale bars, 2 cm.

Delayed floral organ abscission and senescence are generally considered undesirable traits in crops due to their negative impact on reproductive efficiency and yield. However, in ornamental species, particularly in cut flowers, these traits represent key breeding goals, as they directly enhance postharvest longevity, and aesthetic value. Given the conserved roles of BOP proteins in regulating floral organ abscission, we next investigated whether *BOP* genes play repurposed roles in ornamental horticultural plants. To this end, we conducted a BLAST search for homologous BOP proteins in a broad spectrum of commercially important horticultural species. Conserved BOP homologs were detected in multiple taxa, including *Petunia hybrid* (*Ph*), *Catharanthus roseus* (*Cr*), *Tagetes erecta L*. (*Te*), *Rosa chinensis* (*Rc*), *Antirrhinum majus* (*Am*), *Helianthus annuus* (*Ha*), and *Torenia fournieri* (*Tf)* (Figure 6A). Among them, petunia stands out as a well‐established model for studying floral organ development and senescence. Importantly, petunia, like tomato, belongs to the Solanaceae family, and contains three *PhBOP* genes, *PhBOP1*, *PhBOP2*, and *PhBOP3*, which are orthologous to the tomato *SlBOP* genes (Figure 6A). Using CRISPR/Cas9‐mediated genome editing, we generated multiple independent T_0_ transgenic lines harboring insertion or deletion mutations in individual *PhBOP* loci (Figure 6B). Through genotypic screening of subsequent generations, we successfully isolated homozygous lines for single, double, and triple *Phbop* mutants, including *Phbop2*, *Phbop1/2*, *Phbop1/3*, *Phbop2/3*, and *Phbop1/2/3*. Notably, the overall morphology and architecture of these mutants were comparable to that of the wild‐type (WT), suggesting that *PhBOP* mutations do not adversely affect general vegetative growth or development (Figure S9A). To examine whether *PhBOP* genes contribute to floral organ development, we quantified corolla morphology by measuring corolla length and diameter. The *Phbop2* single mutant showed no significant differences in either corolla length or diameter compared with WT (Figure 6C–E). In contrast, *Phbop1/2* and *Phbop2/3* mutants exhibited a slight increase in corolla diameter, while no significant differences in corolla length were observed (Figure 6C–E). Notably, both *Phbop1/3* and *Phbop1/2/3* mutants displayed marked phenotypic alterations, with corolla diameter increased by approximately 3 mm and corolla length reduced by approximately 11 mm (Figure 6C–E). Consequently, the *Phbop1/2/3* flowers developed a flatter corolla architecture (Figure 6F), potentially enhancing their aesthetic value. These findings suggest that *PhBOP* genes act redundantly in regulating corolla morphogenesis, with *PhBOP1* and *PhBOP3* contributing more prominently to these traits.

We then assessed the impacts of *PhBOP* mutations on flower longevity. The day of flowering was designated as day 0 (D0), and floral lifespan was defined as the duration of corolla retention. *Phbop2* and *Phbop1/2* mutants displayed floral lifespans comparable to the wild type (WT) (Figure 6G,H). In contrast, *Phbop2/3* flowers exhibited an approximate 1‐day extension, while *Phbop1/3* and *Phbop1/2/3* mutants showed a pronounced delay of approximately 5 days (Figure 6G,H; Figure S9B). Notably, floral organ abscission was completely suppressed in both *Phbop1/3* and *Phbop1/2/3* mutants (Figure S9B). Similar lifespan extensions were observed in pollinated flowers of these genotypes (Figure S9C,D), indicating that the delay is independent of fertilization and is governed by intrinsic regulatory mechanisms controlling floral organ senescence and abscission. These results suggest that *PhBOP1* and *PhBOP3* play a primary role in regulating floral organ abscission and senescence. Moreover, *Phbop1/2/3* mutant lacked pollen and consequently failed to produce seed pods (Figure S9E,F), suggesting that the three *PhBOP* genes redundantly contribute to pollen development. The absence of pollen may be advantageous for individuals allergic to pollen.

Given that cut flowers constitute a major market category within the commercial floriculture industry, we further assessed the horticultural potential of *PhBOP* mutations using a vase life assay. Freshly cut flowers were placed in water at D0, and their longevity was monitored. WT corollas completely wilted by day 10, whereas *Phbop1/3* flowers remained visibly turgid and retained color (Figure 6I). Continuous monitoring revealed that *Phbop1/3* flowers maintained postharvest freshness for approximately 5 days longer than WT (Figure S9G,H). Collectively, these findings suggest that *PhBOP* mutations can improve ornamental plant traits and address specific market demands, offering a strategic cross‐species translation of conserved functional genes to improve specific traits.

## 3 Conclusion

In response to global climate change and diversified market demands, breeding objectives have become increasingly specialized across crop types. While crops prioritize yield and stress resistance, ornamental plants emphasize traits like flower longevity, color, and morphology. This divergence challenges traditional definitions of beneficial traits. As genetic diversity continues to decline due to intensive selection and the narrowing of germplasm resources, identifying and repurposing genes that are deleterious in crops but potentially beneficial in other plant species presents a promising strategy to mitigate genetic bottlenecks. In this study, we demonstrated the regulatory mechanism of BOP proteins controlling floral organ abscission and senescence in tomato, a model crop species with well‐established research systems. We then translated this functional knowledge to petunia, a representative ornamental horticultural plant, where targeted manipulation of BOP orthologs successfully prolonged floral longevity, and optimized corolla morphology, enhancing ornamental quality. Our study exemplifies how mechanistic insights from crops can inform horticultural breeding and the strategic repurposing of traits traditionally considered detrimental.

Despite the conserved roles of *BOP* genes in floral organ abscission and senescence, higher‐order *Slbop* mutants in tomato and *Phbop* mutants in petunia exhibit divergent phenotypic outcomes. Previous work demonstrated that altered interactions among specific *cis*‐regulatory elements drive evolutionary divergence in *BOP* expression, leading to partial loss of redundancy and altered floral development in *Capsella rubella* compared to *Arabidopsis thaliana* [36]. To explain the phenotypic inconsistency between tomato and petunia *bop* mutants, we investigated the duplication patterns and evolutionary history of the *BOP* gene family across tomato, potato (*Solanum tuberosum*), eggplant (*Solanum melongena*), pepper (*Capsicum annuum*), petunia (*Petunia hybrida*), coffee (*Coffea canephora*), and grape (*Vitis vinifera*). Comparative genomic analyses revealed that most Solanaceae species harbor three BOP homologs located on two chromosomes, with BOP2 and BOP3 forming tandem duplicates (this configuration could not be definitively confirmed in pepper and petunia due to incomplete genome assemblies) (Figure S10A,B). In contrast, grape and coffee, included as outgroups, each possess a single *BOP* gene (Figure S10A,B). Integrated with species phylogeny (Figure S10B), these data indicate that *BOP* gene expansion occurred prior to petunia emergence. Synteny analyses further showed strong collinearity among tomato, potato, and eggplant *BOP* loci (Figure S10C). In tomato and pepper, *SlBOP1* and *SlBOP3* correspond to *CaBOP1*, while *SlBOP2* and *SlBOP3* correspond to *CaBOP2* (Figure S10C). Notably, *SlBOP1* and *SlBOP3* also exhibited conserved synteny with *PhBOP1* in petunia (Figure S10C). These results suggest that *PhBOP2* likely originated from a tandem duplication of an ancestral *BOP3*. Gene duplication is often accompanied by functional divergence. We speculate that *BOP* genes have undergone lineage‐specific specialization, with *PhBOP1* and *PhBOP3* playing dominant roles in floral organ senescence and abscission in petunia, whereas *PhBOP2* contributes minimally to these processes and instead functions redundantly with *PhBOP1/3* in pollen development. In contrast, all three tomato *BOP* genes participate in floral organ senescence and abscission, with *SlBOP2* assuming a predominant regulatory role. Collectively, the differing phenotypic severities observed in higher‐order *bop* mutants of tomato and petunia likely reflect distinct evolutionary trajectories of post‐duplication functional divergence, underscoring that the selection of orthologs for cross‐species breeding should consider lineage‐specific functional specialization.

Beyond the genetic evidence, we demonstrated that SlBOP proteins function via phase separation and physically interact with the ALOG transcription factor TFAM1 to form heterotypic condensates. Recent studies have highlighted the role of biomolecular condensates in regulating diverse developmental processes through phase separation‐mediated compartmentalization of signaling and cellular components [29, 37]. Both SlBOP and TFAM1 proteins harbor conserved cysteine residues (Figures S11A and S12), which in their respective homologs have been shown to undergo oxidation by reactive oxygen species [31, 35, 38], ubiquitous byproducts of plant cellular metabolism that also act as signaling molecules responsive to developmental and environmental stimuli. Given that reactive oxygen species (ROS) have been implicated in regulating floral organ abscission [10, 13], we speculate that the sequential assembly of SlBOPs‐TFAM1 condensates may be driven by ROS‐mediated disulfide bond formation. Similarly, PhBOP proteins also exhibit conserved cysteine residues within the BTB/POZ domain (Figure S11A), previously associated with redox‐responsive phase separation in NPR1 [35], as well as IDRs (Figure S11B). In parallel, BLAST analysis identified PhLSH2 as the petunia ortholog of TFAM1 (Figure S13A) [39], and conserved cysteine residues were likewise found in its ALOG domain, analogous to TMF (Figure S13B), which forms ROS‐sensitive condensates [31]. These findings indicate that PhBOP and PhLSH2 proteins may likewise undergo phase separation and form heterotypic condensates to regulate corolla abscission and senescence. This condensate‐based regulatory module appears to be evolutionarily conserved across species, offering more opportunities for improving ornamental traits through precision breeding. Based on the extended floral longevity observed in tomato *tfam1 Slbop1/2/3* quadruple mutant compared to *Slbop1/2/3* triple mutant (Figure 5C–E), we hypothesize that *PhLSH2* mutation may further prolong flower longevity beyond that observed in *Phbop1/2/3* mutant through conserved pathways regulating floral senescence.

Interestingly, while TFAM1 alone forms nuclear‐localized condensates, BiFC analysis revealed that heterotypic condensates formed by TFAM1 with SlBOP1 or SlBOP2 simultaneously localize to the nucleus and cytoplasm (Figure 4D). This differential subcellular distribution may be related to distinct fusion capacities or oligomerization behaviors between SlBOPs and TFAM1. Co‐IP assays revealed that SlBOP1 and SlBOP2 form oligomers and can be pulled down by TFAM1 (Figure 4C). In contrast, SlBOP3, which predominantly interacts with TFAM1 in the nucleus and exhibits stronger fusion capacity with TFAM1 in vitro compared to SlBOP1 and SlBOP2 (Figure 4D–F), may support the formation of more stable and transcriptionally active condensates for gene regulatory activity in the nucleus. These biochemical differences may underlie the observed unequal genetic redundancy between SlBOP family members and TFAM1 in regulating floral organ abscission and senescence. Given the diverse developmental roles of BOP proteins and the growing understanding of LLPS in plant biology, further investigation is needed to elucidate the functional mechanisms and biological significance of BOP transcriptional cofactors in planta. A deeper understanding of these mechanisms will enable more precise manipulation of phase separation properties to fine‐tune developmental outcomes. This could open up new possibilities for the breeding of fruit and vegetable crops, as well as ornamental horticultural plants.

## 4 Experimental Methods

### 4.1 Plant Materials and Growth Conditions

The tomato (*Solanum lycopersicum*) cultivar M82 was used in this study. The *tfam1*, *Slbop1*, *Slbop2*, *Slbop3*, *Slbop1/2*, *Slbop1/3*, and *Slbop1/2/3* mutants were shared by Z. B. Lippman (Cold Spring Harbor Laboratory). The higher‐order mutants for *tfam1 Slbop1, tfam1 Slbop3, tfam1 Slbop1/2, tfam1 Slbop2/3*, *and tfam1 Slbop1/2/3* were generated by crossing *tfam1* mutants with *Slbop* mutants. The homozygotes were genotyped by PCR and sequencing. The *35S:GFP‐TFAM1*, *35S:GFP‐SlBOP2*, *pTFAM1:GUS*, and *pSlBOP3:GUS* transgenic plants were generated in the M82 background by Agrobacterium‐mediated tissue culture, following the protocol described in the previous study [31]. The seeds of petunia (*P. hybrida* ‘W115’, Mitchel diploid) were provided by Y. Guo (Southwest University). The *Phbop2, Phbop1/2, Phbop1/3, Phbop2/3*, *and Phbop1/2/3* mutants were generated in the W115 background by Agrobacterium‐mediated tissue culture as described in a previous study [40].

The tomato seedlings were grown in a growth room with 16 h of light and 8 h of dark at 26°C, 45%–60% relative humidity under LED (Philips Lighting IBRS) light. The tomato plants were grown in a solar greenhouse. The petunia seedlings and plants were grown in a growth room with 16 h of light and 8 h of dark at 23°C, 45%–60% relative humidity under LED (Philips Lighting IBRS) light.

### 4.2 Fluorescence Microscope of Plants

The tomato *35S:GFP‐TFAM1*, *35S:GFP‐SlBOP2* transgenic plants were grown in growth room at 26°C. Detached leaves were imaged using a Zeiss LSM980 confocal microscope with 20×, 40× objectives. For fluorescence imaging of flower organ abscission zone, the tissue was prepared as follows: First, the flower was dissected to remove excess parts, leaving only the region around the abscission zone. Subsequently, the tissue was fixed in 4% paraformaldehyde (PFA) solution and subjected to vacuum infiltration at 0.8–0.9 MPa for 30 min. Following fixation, the tissue was washed three times with phosphate‐buffered saline (PBS). The tissue was then embedded in 6% agarose (VWR Chemicals) and sectioned into 50 µm slices using a Leica VT1200S microtome. Fluorescence imaging was performed using a Zeiss LSM980 confocal microscope equipped with 20× and 40× objectives.

For transient expression of TFAM1 and SlBOP proteins in tobacco leaves, the coding sequences of TFAM1, SlBOP1, SlBOP2 and SlBOP3 were amplified using the primers listed in Supporting Table S1 and separately cloned into *PRI101‐GFP* vector to generate *35S:SlBOP1‐GFP*, *35S:GFP‐SlBOP1*, *35S:GFP‐SlBOP2*, and *35S:GFP‐SlBOP3*, and cloned into *35S:YFP^N^* (*YNE*) and *35S:YFP^C^* (*YCE*) to generate *TFAM1‐YNE*, *TFAM1‐YCE*, *SlBOP1‐YNE*, *SlBOP1‐YCE*, *SlBOP2‐YNE*, *SlBOP2‐YCE*, *SlBOP3‐YNE*, and *SlBOP3‐YCE* for BiFC assay. *N. benthamiana* (tobacco) leaves were infiltrated with Agrobacterium GV3101 containing the corresponding plasmid. After 48 h, detached tobacco leaves were imaged using a Zeiss LSM980 confocal microscope with 20×, 40× objectives. GFP fluorescence was excited at 488 nm and detected at 500–540 nm. YFP fluorescence was excited at 514 nm and detected at 525–565 nm.

### 4.3 Protein Structure Prediction and Phylogenetic Analysis

The analysis of IDRs was conducted using the “VSL2” algorithm from the “Predictor of Natural Disordered Regions” (PONDR) tool (http://www.pondr.com/). The analysis of RDRs was conducted using the “IUPred2a” algorithm (https://iupred2a.elte.hu/). BTB, DUF, and ANK domains were analyzed by InterPro (https://www.ebi.ac.uk/interpro/). Homologous BOP family proteins across different species were identified using BLASTP within the NCBI BLAST database (https://blast.ncbi.nlm.nih.gov/Blast.cgi). The phylogenetic tree was generated based on protein sequences using MEGA (version 11.0.13).

### 4.4 Recombinant Protein Expression and Purification

The coding sequences of SlBOP proteins fused with GFP were cloned into the *pQE‐80L* vector. The constructs were then transformed into *E. coli Rosetta* (DE3) competent cells, and positive bacteria cultured in LB medium were induced with 0.5 mM isopropyl *β*‐D‐1‐thiogalactopyranoside (IPTG) for 16 h at 14°C. The cells were collected for protein purification using Ni‐NTA (GE Healthcare) affinity beads following standard protocols [31]. The eluted proteins were subjected to buffer exchange and concentration using ultrafiltration tubes (Vivaspin Turbo). Purified proteins for further use were stored in storage buffer (50 mM Tris‐HCl, 200 mM NaCl, pH 7.4) at –80°C after quick freezing in liquid nitrogen for further use.

### 4.5 Phase Separation and FRAP Assay in vitro

For phase separation assays, the purified proteins were centrifuged at 4°C, 14 000 *g* for 10 min and then the supernatants were transferred into new tubes. The purified proteins were subsequently diluted into buffer containing 50 mM Tris‐HCl (pH 7.4) and varying concentrations of NaCl to the indicated final concentrations in the figures. To generate a phase diagram, the diluted solution of phase‐separated protein solution was incubated for 15 min at room temperature in a 384‐well plate. To perform cross‐mixing phase separation assays for mCherry‐TFAM1 and GFP‐SlBOP proteins in vitro, purified proteins (50 mM Tris‐HCl, pH 7.4; 25 mM NaCl; 15 µM protein concentration) were thoroughly mixed and incubated for 15 min at room temperature in a 384‐well plate. Images were captured using a confocal microscope (Leica SP5) with 20×, 40× objectives. GFP fluorescence was excited at 488 nm and detected at 500–540 nm.

For in vitro FRAP analysis, phase‐separated droplets were prepared in 384‐well plates and imaged using a Nikon A1R+ microscope equipped with a 40× objective. Droplets were photobleached with a 488 nm laser pulse, and fluorescence recovery was recorded over the indicated time period.

### 4.6 Yeast Two‐Hybrid Assays

The coding sequences of TFAM1 and SlBOPs were cloned into *pGBD* and *pGAD* vectors, respectively. The SlBOP1‐AD, SlBOP2‐AD, SlBOP3‐AD, and TFAM1‐BD constructs were co‐transformed into the yeast strain AH109, and the yeast two‐hybrid assay was conducted according to a previously described protocol [41].

### 4.7 Co‑Immunoprecipitation and Immunoblotting Assays

The Co‐immunoprecipitation (Co‐IP) assays were conducted as previously described [41]. The coding sequences of TFAM1 were cloned into the *pRI101‐GFP* vector, and SlBOP1, SlBOP2, and SlBOP3 were cloned into the *pCAMBIA1307‐HA‐Flag* vector. These constructs were co‐transfected into tobacco leaves as indicated, and the leaves were harvested after 48 h. Total proteins were extracted using lysis buffer (10 mM Tris‐HCl, 150 mm NaCl, 0.5% NP40) supplemented with 5 mM DTT. The samples for the co‐immunoprecipitation assay were incubated with GFP‐Nanoab‐Agarose beads (Lablead). Proteins were detected via immunoblotting using anti‐GFP (Easybio) and anti‐HA (Sigma–Aldrich) antibodies, respectively.

### 4.8 GUS Staining

Upstream 3 kb DNA fragments of *TFAM1* or *SlBOP3*, and *GUS* were cloned into the *pGWB401* vector. These constructs were introduced into M82 by Agrobacterium‐mediated transformation. The T1 transgenic plants were used for beta‐glucuronidase (GUS) staining. Flowers of *pTFAM1:GUS* and *pSlBOP3:GUS* transgenic plants were immediately immersed in GUS staining solution (0.1 m Na_3_PO_4_, 10 mm EDTA, 0.1% Triton X‐100, 1 mm K_4_[Fe(CN)_6_], 2 mMm X‐Gluc) and subjected to vacuum infiltration at 0.8–0.9 MPa for 30 min. Subsequently, the samples were incubated at 37°C for 12 h. Decolorization was performed using a graded ethanol series (10%, 30%, 50%, 70%, and 100%), with each step lasting 1 h. The stained samples were imaged by ZEISS stereomicroscope stereoscope (SteREO Discovery, v.12).

### 4.9 Scanning Electron Microscopy

For scanning electron microscopy analysis, sepals, and petals were excised from the flower using forceps in liquid nitrogen. Subsequently, the tissues were subjected to critical point drying, mounted onto steel stubs, coated with gold palladium, and observed using scanning electron microscope (HITACHI S‐3000N&Quorum PP3000T).

### 4.10 Histological Analysis

Fresh flowers were fixed in FAA solution (50% [v/v] ethanol, 3.7% [v/v] formaldehyde, and 5% [v/v] acetic acid) under vacuum at 0.8–0.9 MPa for 30 min. The samples were dehydrated through a graded ethanol series (30%–100%, v/v) and incubated in 100% ethanol for 1 h. Dehydrated tissues were embedded using Technovit 7100 resin (Kulzer, Germany), following the manufacturer's instructions. Serial sections (5 µm thick) were prepared using a rotary microtome (Leica RM2265, Germany), stained with 0.25% (w/v) toluidine blue, and observed under a light microscope (LeicaDFC7000T, Germany).

### 4.11 Transcriptional Activity Assays

The GUS–LUC dual reporter system as previously described was used to perform transcription activity assays in vivo [42]. Briefly, a reporter construct containing the *LUCIFERASE* gene driven by a 1.8 kb upstream promoter fragment of *SlATH1* was generated in the pGreenII0800‐LUC vector, in which the *Renilla luciferase* (*REN*) gene under the control of the *CaMV 35S* promoter served as an internal control. GFP‐tagged TFAM1, SlBOP, or IDR‐deleted variant proteins were used as effector constructs. Reporter and effector plasmids were co‐infiltrated into tobacco (*Nicotiana benthamiana*) leaves. Leaf tissues were collected 60 h post‐infiltration and immediately frozen in liquid nitrogen. LUC and REN activities were quantified using the Dual‐Glo Luciferase Assay System (Promega, E1910) following the manufacturer's instructions. Total proteins of leaf samples were extracted with 100 mL ‘Passive Lysis Buffer’ followed by 15 min of 12 000 rpm centrifugation at 4°C. Chemiluminescence signals were subsequently recorded using a GloMax 96 Microplate Luminometer (Promega) with a 2 s delay and a 10 s measurement time for LUC and REN determination. Transcriptional activity was calculated as the LUC/REN ratio.

## Author Contributions

C.X. and X.H. designed and supervised the research. N.X. performed most of the experiments, analyzed the data and prepared the figures. Q.L. performed qRT‐PCR, dual‐luciferase assays, and part of the in vivo phase separation experiments. T.Z. performed the yeast two‐hybrid and co‐immunoprecipitation experiments. Y.Z. performed synteny analysis. Y.X. and D.Y. provided helps in genotyping and plasmid construction. X.H. wrote the manuscript with the input from N.X. All authors have reviewed and approved the final version of the paper.

## Funding

This study was supported by the Beijing Rural Revitalization Agricultural Science and Technology Project (grant number NY2401080000), National Natural Science Foundation of China (grant number 32270371) and the Youth Innovation Promotion Association of the Chinese Academy of Sciences (grant number 2022094).

## Conflicts of Interest

The authors declare no conflicts of interests.

## Supporting information




**Supporting File**: advs75004‐sup‐0001‐SuppMat.docx.

## Data Availability

The data that support the findings of this study are available from the corresponding author upon reasonable request.
