# Supplementary material for: BLADE‐ON‐PETIOLE Genes Enable Genetic Bottleneck Mitigation Through Cross‐Species Repurposing of Floral Persistence Traits
Source: Adv Sci (Weinh). 2026 Mar 28;13(32):e17112. doi: 10.1002/advs.202517112 (PMC13252624; doi:10.1002/advs.202517112)
Supplement: Supplementary file 1 — Supporting File: advs75004‐sup‐0001‐SuppMat.docx. [file ADVS-13-e17112-s001.docx]

**Supplementary data**

**Supplementary Figure S1.** The process of floral organ abscission and senescence in WT.

**Supplementary Figure S2.** Expression analysis of *SlBOP* in AZ tissues. Expression

**Supplementary Figure S3.** Structure analysis of SlBOP proteins.

**Supplementary Figure S4.** SlBOP proteins showing phase separation.

**Supplementary Figure S5.** SlBOP protein forms homodimers in the nucleus and cytoplasm.

**Supplementary Figure S6.** TFAM1 protein is essential for the formation of abscission zone.

**Supplementary Figure S7.** Phylogenetic analysis of ATH1 homologs in tomato.

**Supplementary Figure S8.** Deletion of the IDR2 attenuates puncta formation of the SlBOP2 protein.

**Supplementary Figure S9.** Mutations of the *PhBOP* genes extend the flowering duration in petunia.

**Supplementary Figure S10.** Comparative genomic analysis of the *BOP* genes.

**Supplementary Figure S11.** Sequence alignment and IDR prediction of PhBOP proteins from petunia.

**Supplementary Figure S12.** Sequence alignment of ALOG family proteins in tomato.

**Supplementary Figure S13.** Phylogenetic and sequence alignment of ALOG family proteins.

**Supplementary Table S1.** Primers used in this study.


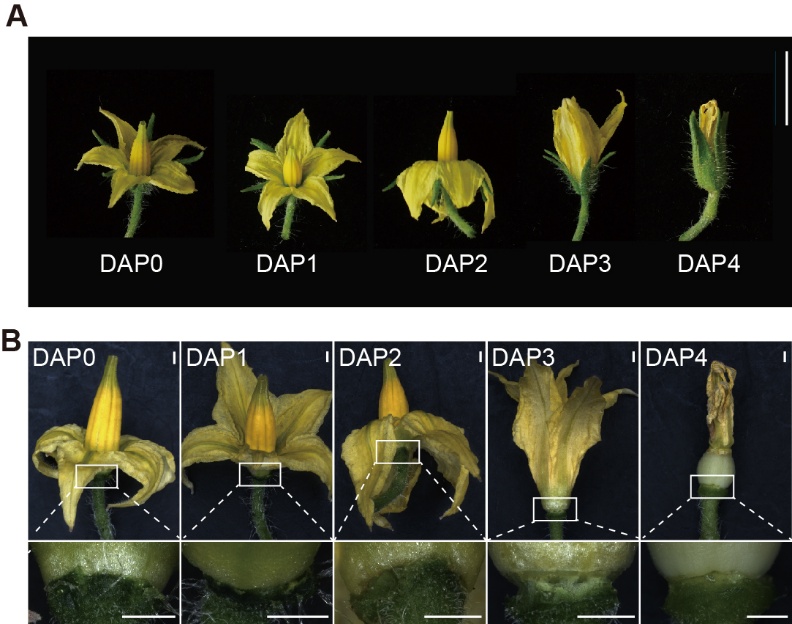


**Figure S1.** The process of floral organ abscission and senescence in WT. A) Representative images of flowers at 0-4 days after pollination (DAP0-4) in WT. Scale bar, 1 cm. B) Representative stereomicroscope images showing the process of floral organ abscission and senescence in WT. Sepals of the flowers were forcibly removed. Scale bars, 1 mm.


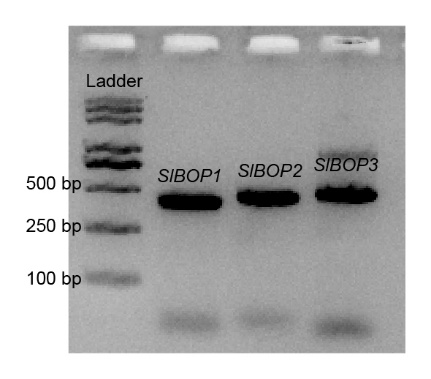


**Figure S2.** Expression analysis of *SlBOP* in AZ tissues. Semi-quantitative RT-PCR of *SlBOP* transcripts in AZ tissues from flowers at DAP 0. DNA ladder is shown to the left.


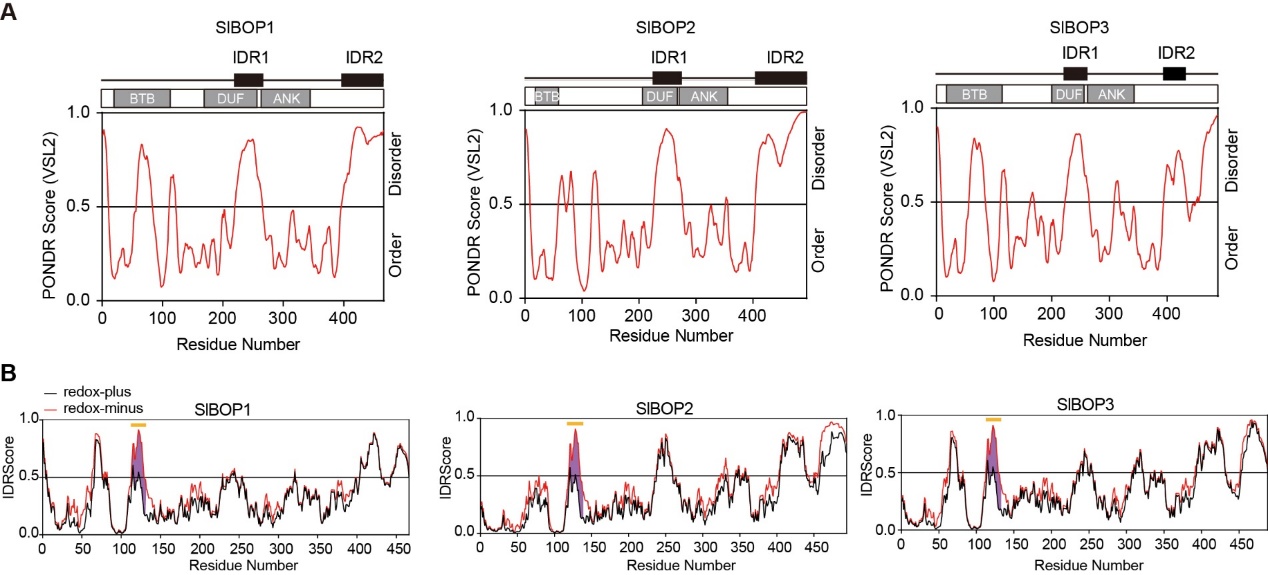


**Figure S3.** Structure analysis of SlBOP proteins. A) Schematic showing protein structure and predicted IDRs of SlBOP proteins. BTB, BTB/POZ domain (Pfam: 00651); DUF, DUF3420 domain (Pfam:11900); ANK, ankyrin repeats (Pfam: 12796). B) Schematics showing predicted redox-sensitive disordered regions (RDRs) of SlBOP proteins. The values represent the predicted disorder scores for each wild-type cysteine (redox plus) or mutated cysteine (redox minus). The size and location of the inferred RDRs are indicated in orange (purple regions), with disorder scores greater than 0.5.


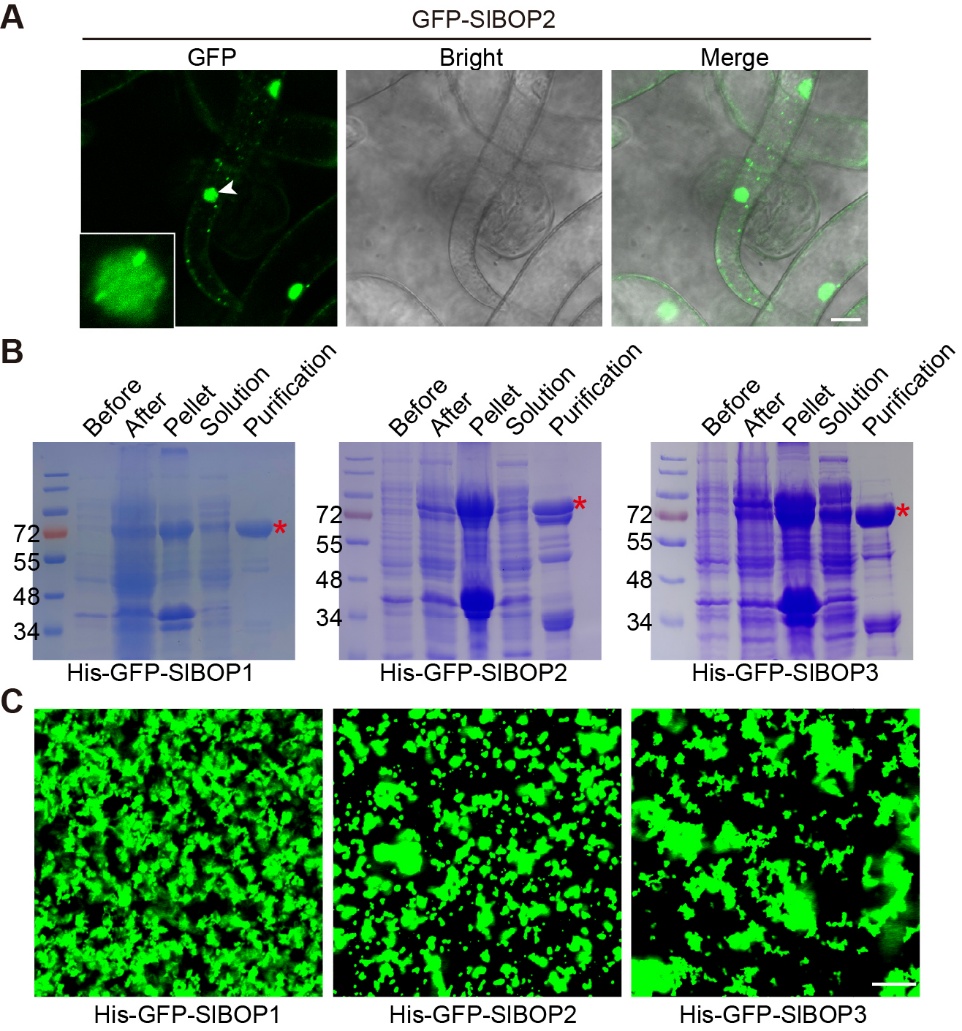


**Figure S4.** SlBOP proteins showing phase separation. A) Images showing SlBOP2 condensates in the leaf epidermal trichomes of tomato transgenic plants expressing GFP-SlBOP2. Scale bar, 10 μm. B) SDS-PAGE gels showing the induction and purification for His-GFP-SlBOP fused proteins. The red stars note recombinant expressed proteins, respectively. C) Microscopy images of phase separation for His-GFP-SlBOP proteins used in this study. Proteins concentration, 15 μM. NaCl concentration, 25 mM. Scale bar, 20 μm.

**
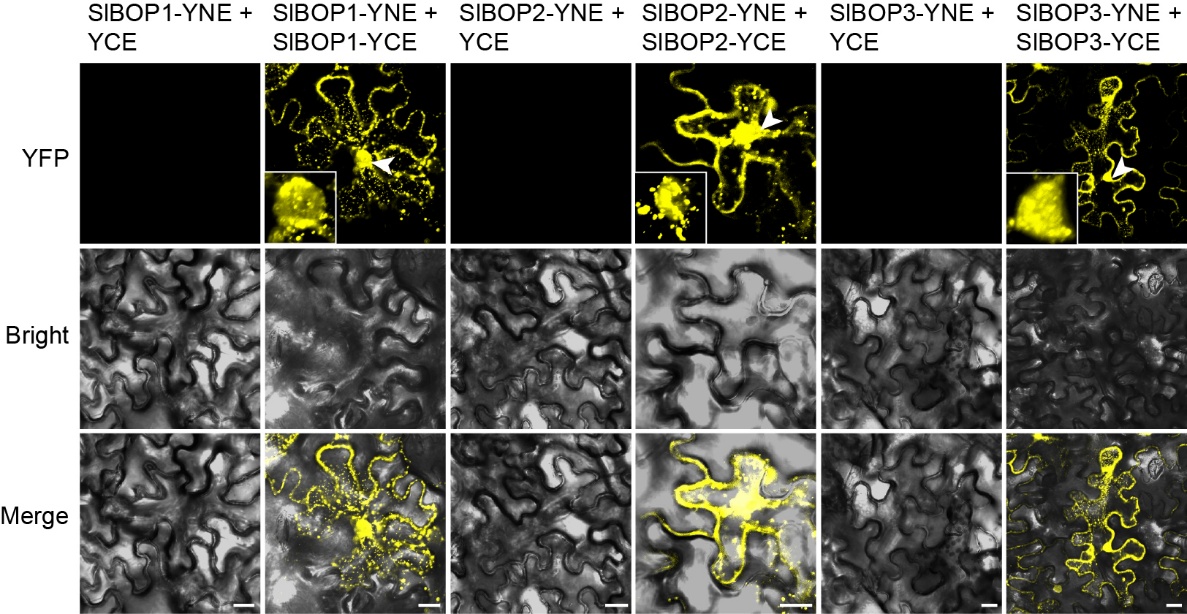
**

**Figure S5.** SlBOP protein forms homodimers in the nucleus and cytoplasm. BiFC assays showing SlBOP proteins form homodimers with punctate localization in both the nucleus and cytoplasm of tobacco leaves, respectively. Scale bars, 20 μm.


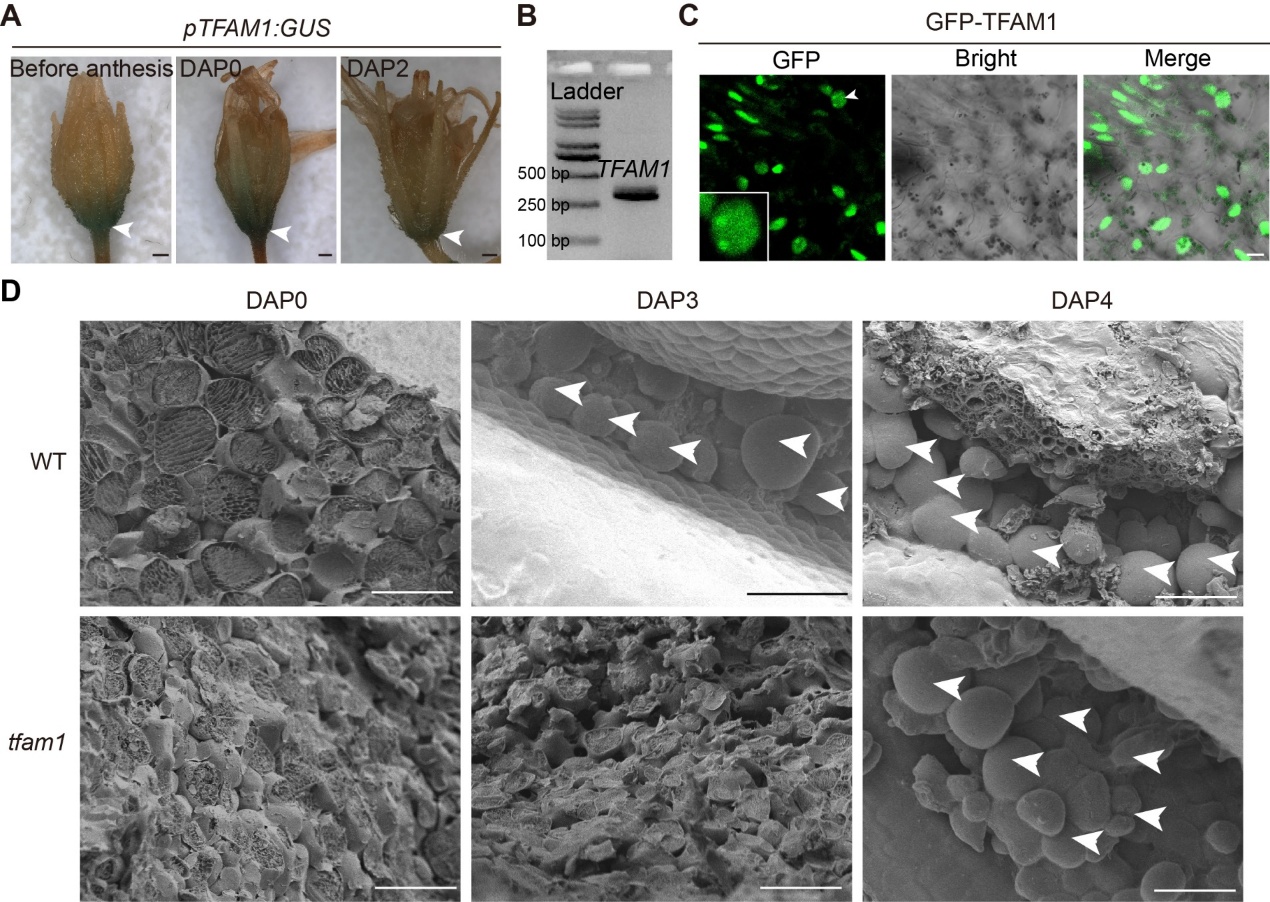


**Figure S6.** TFAM1 protein is essential for the formation of abscission zone. A) Histochemical GUS staining of flowers before anthesis, on the day of pollination (DAP0) and 2 days after pollination (DAP2) from *pTFAM1:GUS* transgenic plants. Scale bars, 1 mm. White arrows indicate petal AZs. B) Semi-quantitative RT-PCR of *TFAM1* transcripts in AZ tissues from flowers at DAP 0. DNA ladder is shown to the left. C) Images showing the TFAM1 condensates in the abscission zone of tomato transgenic plants expressing GFP-TFAM1. Scale bar, 10 μm. D) Scanning electron micrographs of the fracture planes on the receptacle with petals removed in WT and *tfam1* mutants at the DAP0, DAP3 and DAP4. The arrows indicate the spherical elongated cells. DAP, days after pollination. Scale bar, 50 μm.


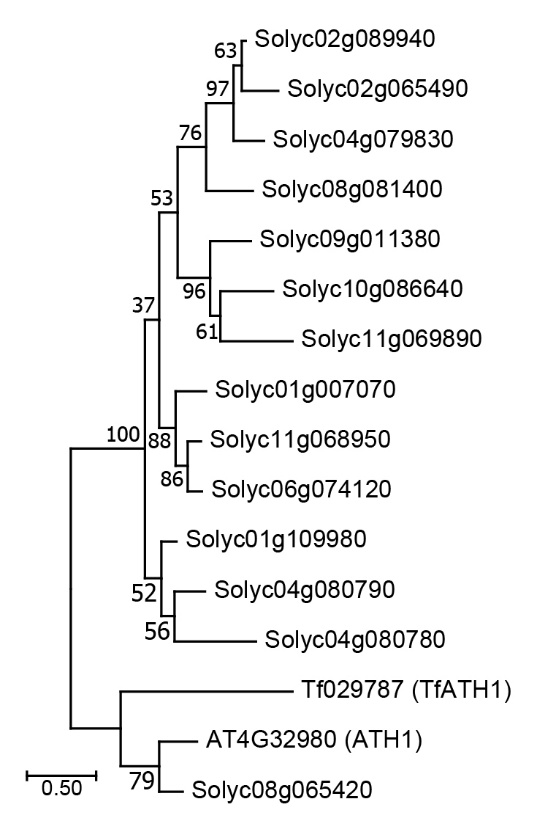


**Figure S7.** Phylogenetic analysis of ATH1 homologs in tomato. Maximum likelihood phylogenetic tree of ATH1, TfATH1 and ATH1 homologs in tomato.


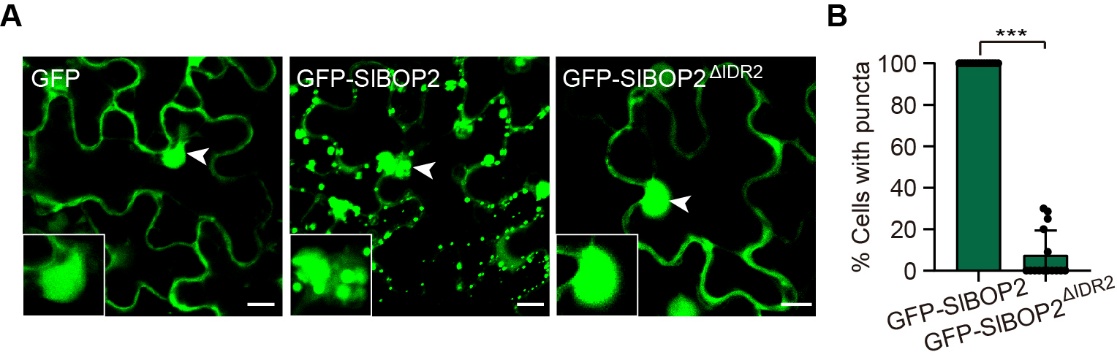


**Figure S8.** Deletion of the IDR2 attenuates puncta formation of the SlBOP2 protein. A) Representative confocal images of GFP, GFP-fused SlBOP2 protein, and its IDR2-deletion variant expressed in tobacco leaves. Scale bars, 10 μm. B) Quantification of the proportion of cells exhibiting condensates for SlBOP2 protein and its IDR2-deleted variant. Data are means ± SD (n = 15, 15, ****P* < 0.001, Student *t*-test). Three independent experiments with similar results were carried out.


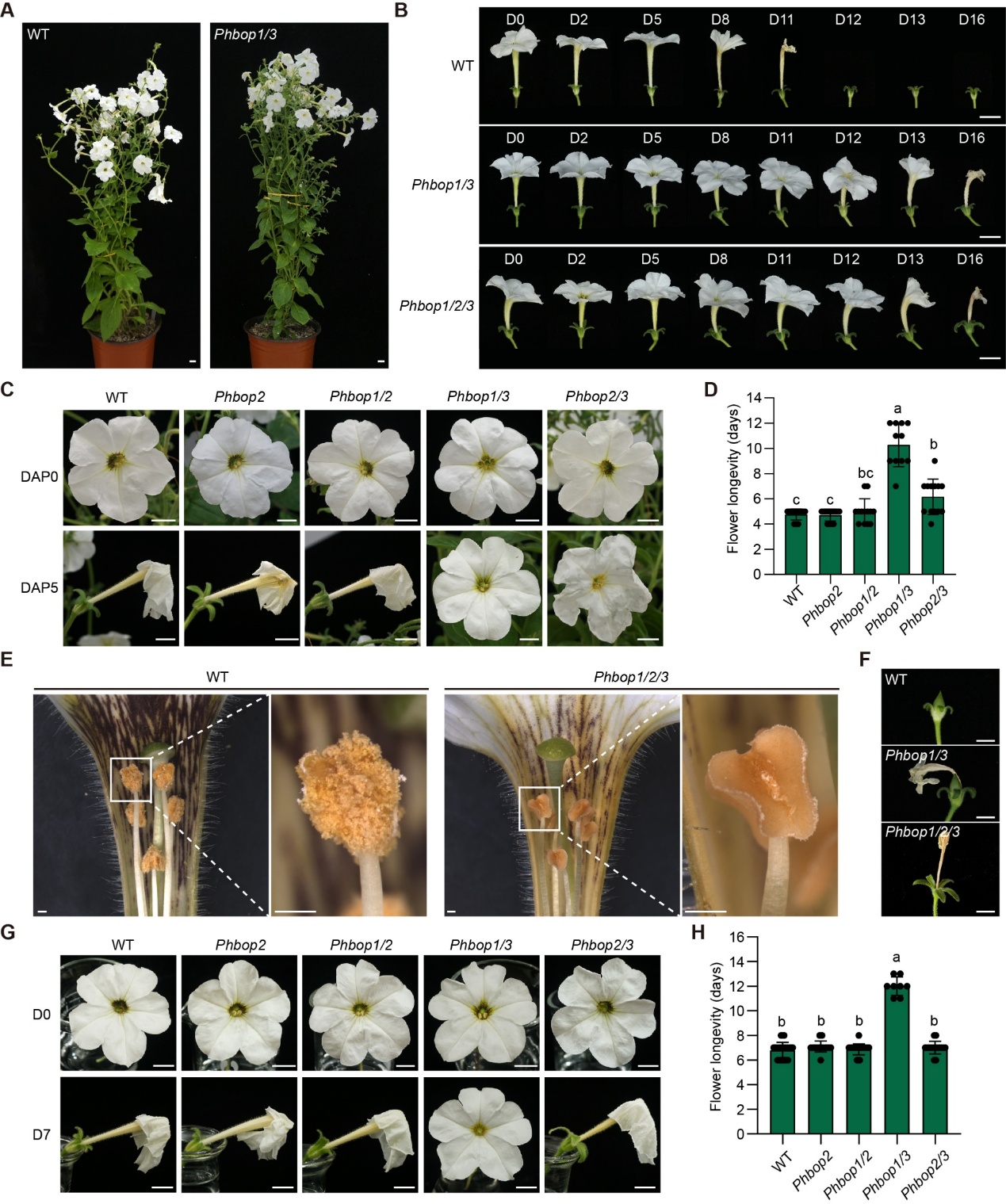


**Figure S9.** Mutations of the *PhBOP* genes extend the flowering duration in petunia. A) Representative plants of WT and *Phbop1/3* mutants. Scale bars, 2 cm. B) Representative images showing the process of corolla abscission and senescence in WT, *Phbop1/3* and *Phbop1/2/3* mutants. Numbers indicate the days after anthesis (D0-16). Scale bars, 2 cm. C) Representative pollinated flowers from WT and *Phbop* mutants at the day of pollination (DAP0) and 5 days after pollination (DAP5). Scale bars, 1 cm. D) Quantification of the flower duration time of pollinated flowers in WT and *Phbop* mutants. Data are means ± SD (n = 17, 17, 13, 11, 13), where n represents the number of biologically independent flowers. Statistical analysis was performed using one-way ANOVA followed by Tukey’s multiple comparisons test. Different letters indicate statistically significant differences among groups (*P* < 0.05). E) Representative stamens and pistils from WT and *Phbop1/2/3* mutants. Scale bars, 500 μm. F) Representative images showing pods formation in WT and *Phbop1/3* mutants, and the absence of pod development in the *Phbop1/2/3* mutant, characterized by a bare floral receptacle. Scale bars, 1 cm. G) Representative detached flowers from WT and *Phbop* mutants at the day of anthesis (D0) and 7 days after anthesis (D7). Scale bars, 1 cm. H) Quantification of the flower duration time of detached flowers from WT and *Phbop* mutants. Data are means ± SD (n = 35, 21, 29, 8, 16), where n represents the number of biologically independent flowers. Statistical analysis was performed using one-way ANOVA followed by Tukey’s multiple comparisons test. Different letters indicate statistically significant differences among groups (*P* < 0.05).


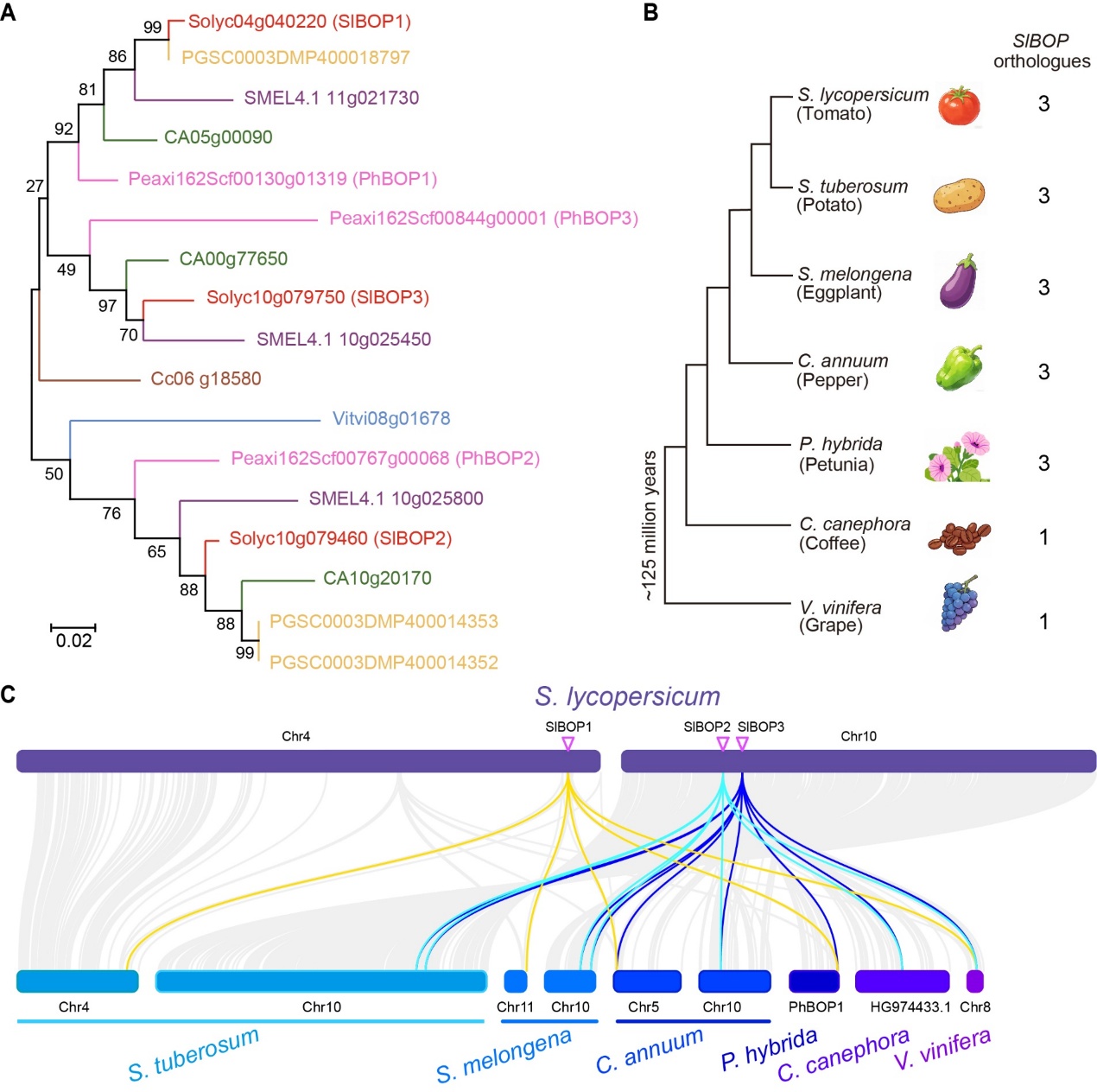


**Figure S10.** Comparative genomic analysis of the *BOP* genes. A) Maximum likelihood phylogenetic tree of BOP proteins in tomato (*Solanum lycopersicum*), potato (*Solanum tuberosum*), eggplant (*Solanum melongena*), pepper (*Capsicum annuum*), petunia (*Petunia hybrida*), coffee (*Coffea canephora*) and grape (*Vitis vinifera*). B) Copy number variation of SlBOP orthologues in the Solanaceae, coffee and grape. C) Synteny analysis of *BOP* loci in tomato (*Solanum lycopersicum*), potato (*Solanum tuberosum*), eggplant (*Solanum melongena*), pepper (*Capsicum annuum*), petunia (*Petunia hybrida*), coffee (*Coffea canephora*) and grape (*Vitis vinifera*).


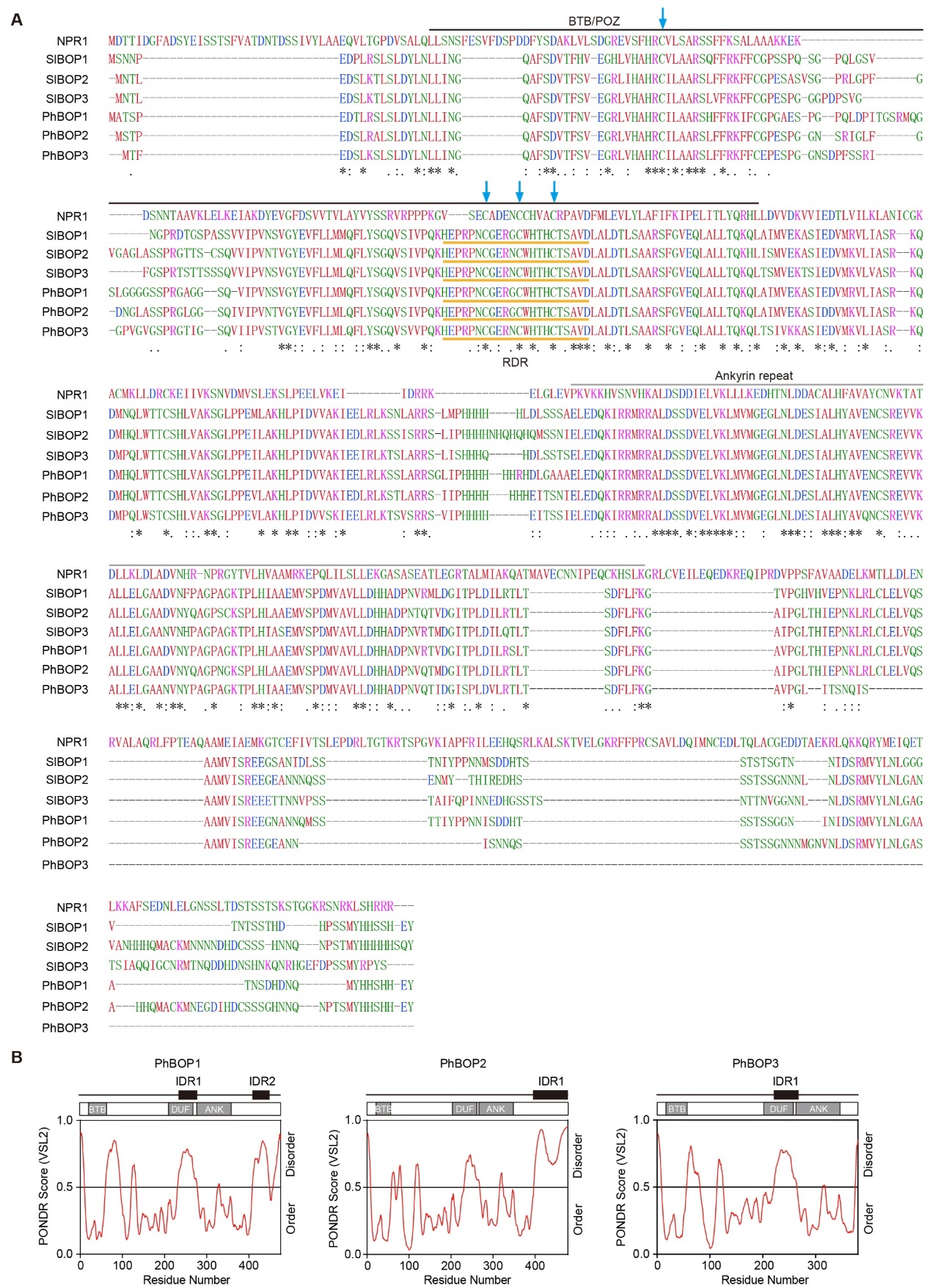


**Figure S11.** Sequence alignment and IDR prediction of PhBOP proteins from petunia. A) Sequence alignment of SlBOP1/2/3, PhBOP1/2/3 proteins with NPR1. Black and grey lines indicate the BTB/POZ and ankyrin repeat domains, respectively. Blue arrowheads indicate the conserved cysteine residues within the BTB/POZ domain shared by SlBOP1/2/3, PhBOP1/2/3 and NPR1. RDR, redox-sensitive IDR. B) Schematic showing protein structure and predicted IDRs of PhBOP proteins. BTB, BTB/POZ domain (Pfam: 00651); DUF, DUF3420 domain (Pfam:11900); ANK, ankyrin repeats (Pfam: 12796).


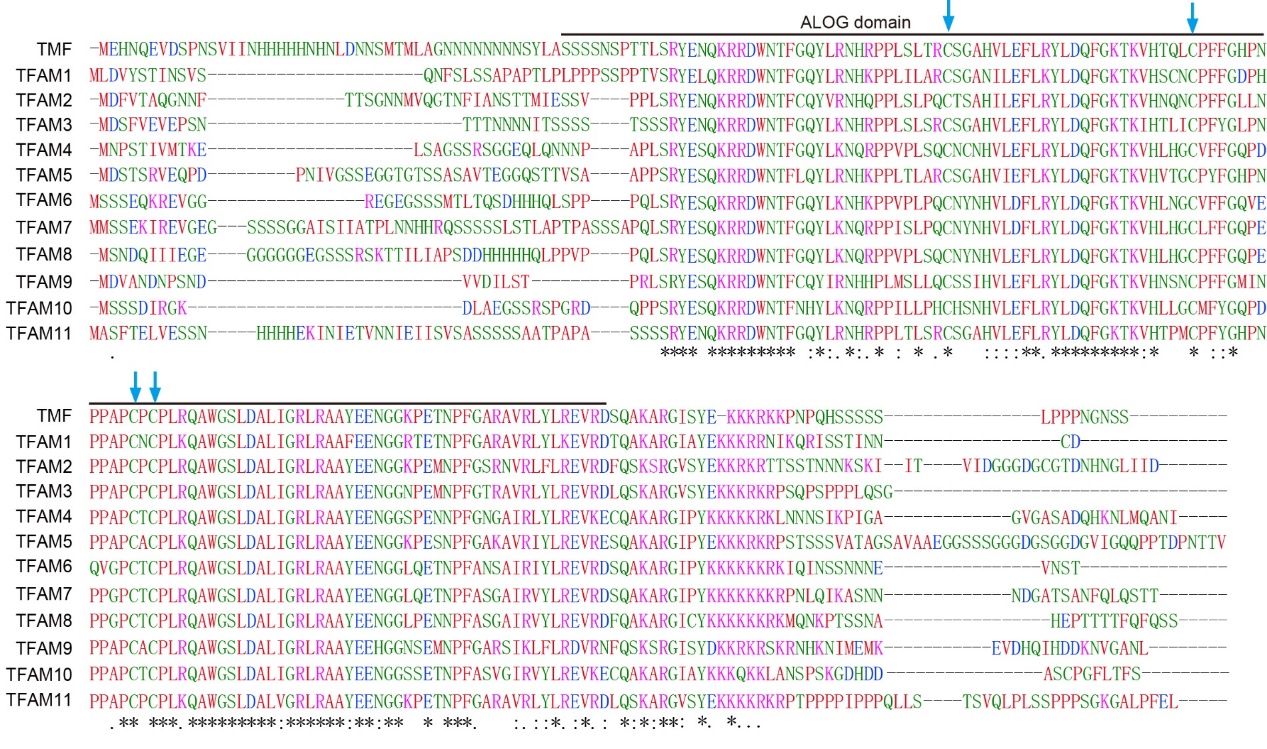


**Figure S12.** Sequence alignment of ALOG family proteins in tomato. The black line indicates the ALOG domain. Blue arrowheads indicate the conserved cysteine residues shared by ALOG family proteins.


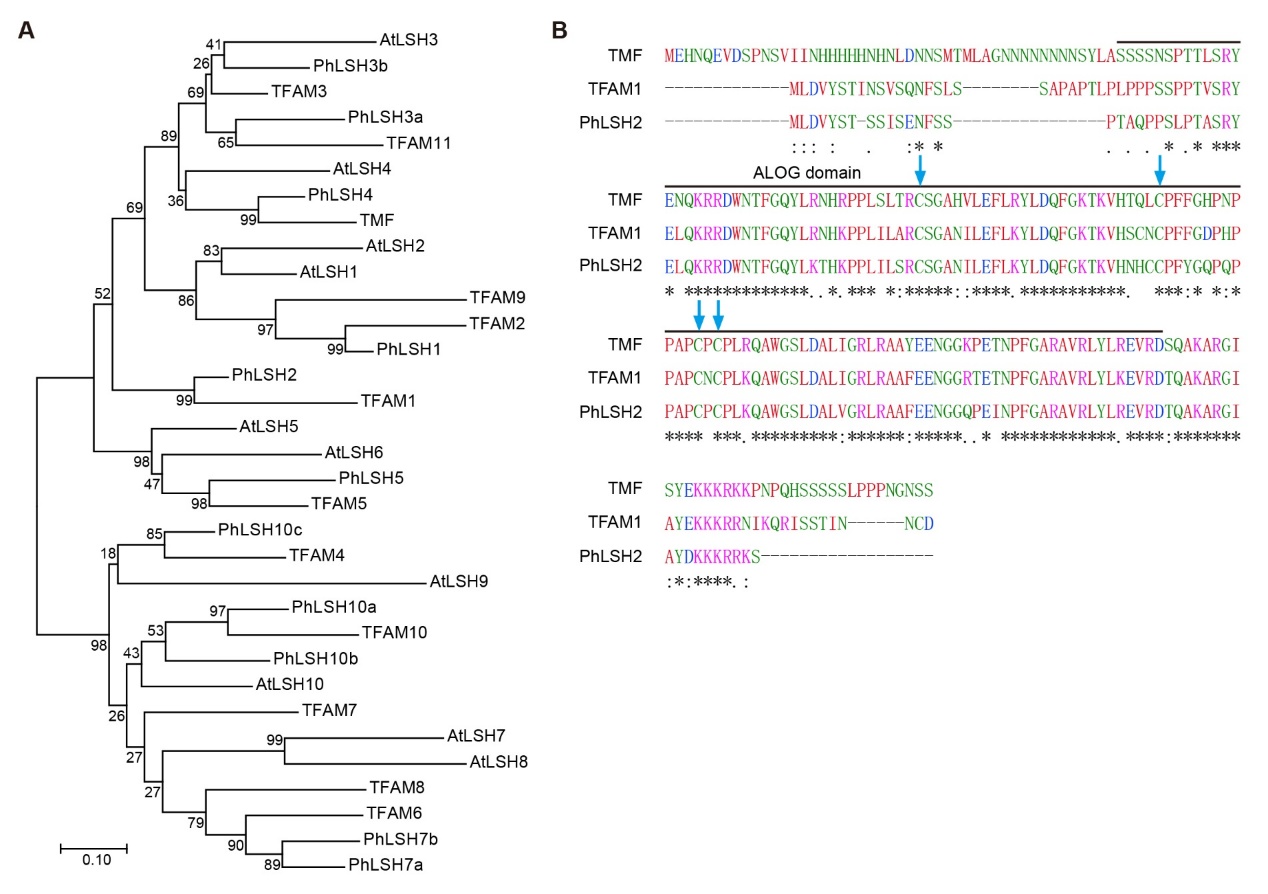


**Figure S13.** Phylogenetic and sequence alignment of ALOG family proteins. A) Neighbour joining phylogenetic tree of ALOG family proteins from *Arabidopsis thaliana* (*At*), *Solanum lycopersicum* (*Sl*) and *Petunia hybrid* (*Ph*). B) Sequence alignment of TFAM1 and PhLSH2 proteins with TMF. Black line indicates the ALOG domain. Blue arrowheads indicate the conserved cysteine residues within the ALOG domain shared by TMF, TFAM1 and PhLSH2.

**Table S1.** A list of primers used in this study.

| Primer name | Primer sequence (5' to 3') | Purpose |
| --- | --- | --- |
| TFAM-1-geno-F | AAAGTAACACAGCCATGTTAGACG | Genotyping |
| TFAM-1-geno-R | GCCGAAAAACGGACAATTAC | Genotyping |
| SlBOP1-geno-F | CACAAGACAAAACACCAACCA | Genotyping |
| SlBOP1-geno-R | GCTCATGTTTTTGCGGTACA | Genotyping |
| SlBOP2-geno-F | GCCACTAACCTTTCTCCTATTG | Genotyping |
| SlBOP2-geno-R | GGCTGATGTGCAATGTGTATG | Genotyping |
| SlBOP3-geno-F | CACAAAATCATCTTCCACTATGTGA | Genotyping |
| SlBOP3-geno-R | TGCAATGTGTATGCCAACAG | Genotyping |
| PhBOP1-T1-F | ATTGGCGAAGATCCTCCTCCGCCG | Constructing |
| PhBOP1-T1-R | AAACCGGCGGAGGAGGATCTTCGC | Constructing |
| PhBOP1-T2-F | ATTGAGGCCTAATTGTGGAGAGAG | Constructing |
| PhBOP1-T2-R | AAACCTCTCTCCACAATTAGGCCT | Constructing |
| PhBOP2-T1-F | ATTGTAGGCCTGTTCGGCGACAAC | Constructing |
| PhBOP2-T1-R | AAACGTTGTCGCCGAACAGGCCTA | Constructing |
| PhBOP2-T2-F | ATTGTGTGAACCACCTAAGCCCCT | Constructing |
| PhBOP2-T2-R | AAACAGGGGCTTAGGTGGTTCACA | Constructing |
| PhBOP3-T1-F | ATTGAGAATAGGCCCCGTGGGCGT | Constructing |
| PhBOP3-T1-R | AAACACGCCCACGGGGCCTATTCT | Constructing |
| PhBOP3-T2-F  PhBOP3-T2-R  PhBOP1-geno-F  PhBOP1-geno-R  PhBOP2-geno-F | ATTGCTTTCCCCACAATTTGGCCT  AAACAGGCCAAATTGTGGGGAAAG  AAATCTTTTGTGGGCCGGGA  AGTGATAAACAAACCTGAGTGAGC  TTCTTTTGTGGGCCGGAGT | Constructing  Constructing  Genotyping  Genotyping  Genotyping |
| PhBOP2-geno-R | TGTGTATGCCAACACCCTCTT | Genotyping |
| PhBOP3-geno-F | TGGTTCATGCACACAGATGC | Genotyping |
| PhBOP3-geno-R | AGAATTCAGATCCCACTGTCCAA | Genotyping |
| pTFAM1-F | CGACGGCCAGTGCCAAGCTTGTATCATT TTTAGCAGAAAAC | Constructing |
| pTFAM1-R | GGCTGTGTTACTTTTGTGTGGG | Constructing |
| GUS-F | CCCACACAAAAGTAACACAGCCATGTTA | Constructing |
|  | CGTCCTGTAGAAACCCC |  |
| GUS-R | GAACGATCGGGGAAATTCGAGCTCTCAC | Constructing |
|  | ACGTGGTGGTGGTGGTG |  |
| pSlBOP3-GUS-F | AAGATCAGAAGTACTGGGGAGTTAGGA | Constructing |
|  | GTAGTGGC |  |
| pSlBOP3-GUS-R | TTCGGATCCGGTACCCTAAACGACCTTC | Constructing |
|  | AACACTAAAA |  |
| 101-GFP-F | CTGTTGATACATATGCCCGTCGACATGGT | Constructing |
|  | GAGCAAGGGCGAGG |  |
| Linker-GFP-R | AAGCTTAGCTCCACCTCCACCTCCAGGC  CGGCCCTTGTACAGCTCGTCCATGC | Constructing |

| Primer name | Primer sequence (5' to 3') | Purpose |
| --- | --- | --- |
| Linker-TFAM1-F | GGAGGTGGAGGTGGAGCTAAGCTTATG | Constructing |
|  | TTAGACGTATATAGTACC |  |
| 101-TFAM1-R | GTTGTTGATTCAGAATTCGGATCCGGTA | Constructing |
|  | CCCTAATCACAATTATTAATAG |  |
| 101-TFAM1-F | CTGTTGATACATATGCCCGTCGACATG | Constructing |
|  | TTAGACGTATATAG |  |
| TFAM1-R | ATCACAATTATTAATAGTAC | Constructing |
| TFAM1-mCherry-F | GTAGTACTATTAATAATTGTGATGGCCG  GCCTGGAGGTGGAGGTGGAGCTAAGC | Constructing |
|  | TTGTGAGCAAGGGCGAGGAGG |  |
| 101-mCherry-R | GATTCAGAATTCGGATCCGGTACCCTA | Constructing |
|  | CTTGTACAGCTCGTCC |  |
| 101-SlBOP1-F | CACTGTTGATACATATGCCCGTCGACAT | Constructing |
|  | GAGTAATAATCCTGAAGATCCC |  |
| SlBOP1-R | ATACTCATGTGATGAATGGTG | Constructing |
| SlBOP1-GFP-F | CACCATTCATCACATGAGTATCCCGGGG | Constructing |
|  | GCCGGCCTGGAGGTGGAGGTGGAGCTA |  |
|  | AGCTTATGGTGAGCAAGGGCGAGG |  |
| 101-GFP-R | GATTCAGAATTCGGATCCGGTACCTTAC | Constructing |
|  | TTGTACAGCTCGTCC |  |
| 101-salI-GFP-F | CTGTTGATACATATGCCCGTCGACATGG | Constructing |
|  | TGAGCAAGGGCGAGG |  |
| 101-SlBOP2-R | GATTCAGAATTCGGATCCGGTACCCTAA | Constructing |
|  | TACTGAGAATGATGATG |  |
| GFP-SlBOP3-F | CGGCATGGACGAGCTGTACAAGGGCCG | Constructing |
|  | GCCTGGAGGTGGAGGTGGAGCTAAGCT |  |
|  | TATGAATACTCTTGAAGATTCC |  |
| 101-SlBOP3-R | GATTCAGAATTCGGATCCGGTACCTCAT | Constructing |
|  | GAATATGGACGATACATC |  |
| PQE-mCherry-F | ATCCGCATGCGAGCTCGGTACCATGGTG | Constructing |
|  | AGCAAGGGCGAGGA |  |
| mCherry-R | CTTGTACAGCTCGTCCATGCCG | Constructing |
| mCherry-TFAM1-F | CATGGACGAGCTGTACAAGGGCCGGCC  TGGAGGTGGAGGTGGAGCTAAGCTTATG | Constructing |
|  | TTAGACGTATATAGTACC |  |
| PQE-TFAM1-R | CCAGCTCAGCTAATTAAGCTTCTAATCAC | Constructing |
|  | AATTATTAATAG |  |
| PQE-SlBOP1-F | GGTGGAGGTGGAGCTAAGCTTATGAGTA | Constructing |
|  | ATAATCCTGAAGATCCC |  |
| PQE-SlBOP1-R | CCAGCTCAGCTAATTAAGCTTTTAATACT | Constructing |
|  | CATGTGATGAATGGTG |  |
| PQE-SlBOP2-F | GGAGGTGGAGGTGGAGCTAAGCTTATGA | Constructing |
|  | ATACTCTTGAAGATTCC |  |

| Primer name | Primer sequence (5' to 3') | Purpose |
| --- | --- | --- |
| PQE-SlBOP2-R | CCAGCTCAGCTAATTAAGCTTCTAATAC | Constructing |
|  | TGAGAATGATGATG |  |
| PQE-SlBOP3-F | GGAGGTGGAGGTGGAGCTAAGCTTATG | Constructing |
|  | AATACTCTTGAAGATTCC |  |
| PQE-SlBOP3-R | CCAGCTCAGCTAATTAAGCTTTCATGAA | Constructing |
|  | TATGGACGATAC |  |
| YNE-TFAM1-F | CCAGGCCTACTAGTGGATCCATGTTAGA  CGTATATAGTAC | Constructing |
| YNE-TFAM1-R | CGGGAGCGGTACCCTCGAGCTAATCAC  AATTATTAATAG | Constructing |
| YNE-SlBOP1-F | GGGCCCAGGCCTACTAGTGGATCCATG  AGTAATAATCCTGAAG | Constructing |
| YNE-SlBOP1-R | CCTACCCGGGAGCGGTACCCTCGAGTT  AATACTCATGTGATG | Constructing |
| YNE-SlBOP2-F | GGGCCCAGGCCTACTAGTGGATCCATG  AATACTCTTGAAGATTCC | Constructing |
| YNE-SlBOP2-R | CCTACCCGGGAGCGGTACCCTCGAGCT  AATACTGAGAATGATGATG | Constructing |
| YNE-SlBOP2-F | GGGCCCAGGCCTACTAGTGGATCCATG  AATACTCTTGAAGATTCC | Constructing |
| YNE-SlBOP3-R | CCTACCCGGGAGCGGTACCCTCGAGTC  ATGAATATGGACGATAC | Constructing |
| 101-BOP2(-IDR2)-R | CAGAATTCGGATCCGGTACCCTATACCA  TAGCCGCGGATTGAAC | Constructing |
| 0800-pBL4-F | TGCAGCCCGGGGGATCAATAACATACT  CAATATATTCGATC | Constructing |
| pBL4-LUC-R | CTAGAACTAGTGGATCTTCTAGTTGAA  ATTTGAGTTCCAC | Constructing |
| qUbi-F | CGTGGTGGTGCTAAGAAGAG | qRT-PCR |
| qUbi-R | ACGAAGCCTCTGAACCTTTC | qRT-PCR |
| SlATH1-qRT-F | GCTTTGTTGCAAGTGGTTGACG | qRT-PCR |
| SlATH1-qRT-R | GATCCAACTCGGTCACAGCA | qRT-PCR |
| SlBOP1-RT-F | GGGCCGAGAGATACTGGTTC | semi-qRT-PCR |
| SlBOP1-RT-R | TTTGGCCAACATTTCGGGTG | semi-qRT-PCR |
| SlBOP2-RT-F | TCCTAGCCAAACACCTCCCT | semi-qRT-PCR |
| SlBOP2-RT-R | ACCGTTTGGGTATTCGGGTC | semi-qRT-PCR |
| SlBOP3-RT-F | ACCTGTGAACTCGGTAGGGT | semi-qRT-PCR |
| SlBOP3-RT-R | CGAACTTAGGTCGTGCTGGT | semi-qRT-PCR |
| TFAM1-RT-F | CACTGATACTTGCGCGATGC | semi-qRT-PCR |
| TFAM1-RT-R | CATAAGCAATCCCCCTCGCT | semi-qRT-PCR |
| UBI-RT-F | GCAGCTTGAGGATGGTCGTA | semi-qRT-PCR |
| UBI-RT-R | AATCGCCTCCAGCCTTGTTG | semi-qRT-PCR |
